# RNA2HLA: HLA-based quality control of RNA-seq datasets

**DOI:** 10.1093/bib/bbab055

**Published:** 2021-03-24

**Authors:** Irina Chelysheva, Andrew J Pollard, Daniel O’Connor

**Affiliations:** Oxford Vaccine Group, Department of Paediatrics, University of Oxford, and the NIHR Oxford Biomedical Research Centre, Oxford, UK; Oxford Vaccine Group, Department of Paediatrics, University of Oxford, and the NIHR Oxford Biomedical Research Centre, Oxford, UK; Oxford Vaccine Group, Department of Paediatrics, University of Oxford, and the NIHR Oxford Biomedical Research Centre, Oxford, UK

**Keywords:** HLA, RNA-sequencing, bioinformatics, transcriptomics, system biology, quality control

## Abstract

RNA-sequencing (RNA-seq) is a widely used approach for accessing the transcriptome in biomedical research. Studies frequently include multiple samples taken from the same individual at various time points or under different conditions, correct assignment of those samples to each particular participant is evidently of great importance. Here, we propose taking advantage of typing the highly polymorphic genes from the human leukocyte antigen (HLA) complex in order to verify the correct allocation of RNA-seq samples to individuals. We introduce RNA2HLA, a novel quality control (QC) tool for performing study-wide HLA-typing for RNA-seq data and thereby identifying the samples from the common source. RNA2HLA allows precise allocation and grouping of RNA samples based on their HLA types. Strikingly, RNA2HLA revealed wrongly assigned samples from publicly available datasets and thereby demonstrated the importance of this tool for the quality control of RNA-seq studies. In addition, our tool successfully extracts HLA alleles in four-digital resolution and can be used to perform massive HLA-typing from RNA-seq based studies, which will serve multiple research purposes beyond sample QC.

## INTRODUCTION

The human leukocyte antigens (HLA) encoded by highly polymorphic genes, located within the major histocompatibility complex (MHC) in humans, play a crucial role in the adaptive immune system. The genes of the HLA region are divided into two main classes, Class I (*HLA-A*, *HLA-B* and *HLA-C*, and others) and Class II (*HLA-DPA1, HLA-DPB1, HLA-DQA1, HLA-DQB1, HLA-DRA, HLA-DRB1* and others). HLA-typing is widely used in the clinical medicine, for example, matching the HLA alleles between the donors and recipients in the stem-cell or organ transplantation [1].

An extensive amount of single-nucleotide polymorphisms (SNPs) within the sequences led to the development of special nomenclature for the alleles encoding HLA genes, where the four-digital resolution is a level of HLA-typing, used for clinical purposes, as it defines a specific HLA protein, and further precision in the typing only shows synonymous substitutions within the coding DNA region. While IPD-IMGT/HLA Database [2] currently contains over 25 000 sequences of known HLA alleles, there are six HLA genes with the highest number of variant alleles—*HLA-A, HLA-B, HLA-C, HLA-DPB1, HLA-DQB1, HLA-DRB*1—comprising nearly 23 000 alleles in total.

Multiple laboratory-based techniques for HLA typing have been established, including serotyping [3], polymerase chain reaction (PCR) with sequence-specific primers (PCR-SSP) [4], typing based on Sanger sequencing (SBT) [5]. However, rapidly growing next-generation sequencing (NGS) technologies reveal the possibility of using computational approaches to perform HLA-typing by capturing HLA genotypes from the sequencing reads.

Nowadays, various computational tools allow HLA-typing using NGS reads from genomic DNA sequencing [6, 7, 8]. RNA-seq technologies produce shorter nucleic acid sequence reads compared with the whole-genome sequencing platforms. Moreover, the expression levels of genes play crucial role in their detection and coverage in RNA-seq samples. Therefore, genotyping of highly polymorphic HLA genes from RNA-seq is a particularly challenging bioinformatics task, since the sequence differed by only a few nucleotides lead to another allele variant. Multiple algorithms have been developed over recent years addressing this issue and high-resolution HLA-typing is now possible for RNA-seq datasets [6, 8, 9, 10, 11, 12].

The enormous amount of possible alleles for each of HLA genes leads to the almost unique combination of HLA alleles for each individual, which explains the difficulty of coupling the donors and recipients for tissue transplantation. 10/10 alleles match (without taking into account HLA-DPB1 alleles) can be found only for 50% of the patients across all the European population [13].

Conversely, in the case of sequencing data, this reveals an exclusive signature of each individual allowing precise allocation of each sequencing sample to a particular individual. A sample identity check is of particular importance for clinical studies, such as in clinical trials or drug testing, which typically includes multiple sequencing samples from the same study participant taken at various time points or under changing conditions. An extensive sample size of such studies, sometimes including hundreds of participants, along with a complex sequencing procedure, frequently involving several different facilities, increases the probability of mislabeling at any point during the sample preparation, sequencing library construction, sequencing itself or data transfer.

Taking together the advantage of the uniqueness of the HLA allele combination for each individual, we propose high-throughput HLA-typing as quality control (QC) for RNA-seq studies, which can be seamlessly integrated into existing bioinformatics pipelines.

Currently, there is no HLA-typing tool, which allows performance of a global study-wide comparison between the RNA-seq samples. None of the previously developed approaches had a purpose of using HLA genotypes for sample identification and matching, thus all of them run over one RNA-seq sample at the time, which would require extensive programming skills involved in the downstream processing to perform the QC and cross-compare hundreds of samples within the study. However, the existing programs and algorithms developed for HLA-typing can serve as a template for the creation of the HLA-based QC tool.

Here, we developed RNA2HLA—novel command-line QC tool performing study-wide HLA-typing on RNA-seq data and matching the samples from the common source based on their HLA types.

## METHODS

### Selection of HLA-typing algorithm

We performed a comparison of open-source HLA-typing programs and evaluated their utility. The general features of widely used HLA-typing programs [6, 8, 9, 10] have been summarized in Table 1 (only the tools allowing RNA-seq samples as input were included for the comparison). While paired-end RNA-seq data can be used for HLA-typing, none of the existing programs clearly state the possibility of defining HLA genotypes based on the single-end RNA-seq sample.

**Table 1:** Overview of HLA-typing programs

Program	Input format		Sequencing type			HLA resolution	Languages	OS
	fastq	compr. fastq	DNA	RNA				
				single-end	paired-end			
HISAT2	+	+	+	-(?)	+	8 digits	C++, Python, JAVA	Unix/Linux
HLAscan	+	−	+	−	+	4 digits	Python	Unix/Linux
seq2HLA	+	+	−	-(?)	+	4 digits	Python, R	Unix/Linux, Mac OS, Windows (?)
HLAforest	+	−	−	−	+	8 digits	Perl	Unix/Linux

Abbreviations: DNA: DNA-seq, RNA: RNA-seq, comp.fastq: compressed fastq,(?): potential to be implemented.

HISAT2 [8] and HLAforest [10] type HLA alleles to the higher, eight-digital, resolution compared to seq2HLA [9] and HLAscan [6], both of which allow HLA genotyping to four digits. Four-digital resolution suitable for the purposes of distinguishing the individuals within particular study, as this provides enough combinations of the six most variable HLA genes encoded by two alleles each (one inherited from each parent), leading to the 12 alleles of HLA genes in total to be included in typing. Based on that, four-digital resolution HLA-typing programs were preferred over the others, as they gained the advantage of a smaller size of reference databases, which improved the HLA-typing speed and reduced memory usage.

HLA-typing for the QC purposes is expected to be done at the very beginning of the bioinformatics pipeline for the processing of the samples, e.g. ahead of the classical mapping to the reference genome. Therefore, the raw sequencing data format—fastq—is the most suitable input for the HLA-based QC tool.

Further, platform-independent tools have an advantage over the others, as they do not require specific knowledge of a particular operating system (OS). Straightforward set up and interface along with a small number of specific dependencies required for HLA-typing served as an additional benchmark.

Based on all the above-mentioned criteria, seq2HLA [9], written in Python and R, has been selected as a template for the creation of an HLA-based QC tool for RNA-seq studies. The underlying HLA-typing algorithm and the assignment of the *P*-values for the confidence of the typing have been taken from seq2HLA; however, the original scripts have been significantly modified and rewritten in order to serve as a core for the novel tool—RNA2HLA.

### Overall workflow of RNA2HLA

**Figure 1:**
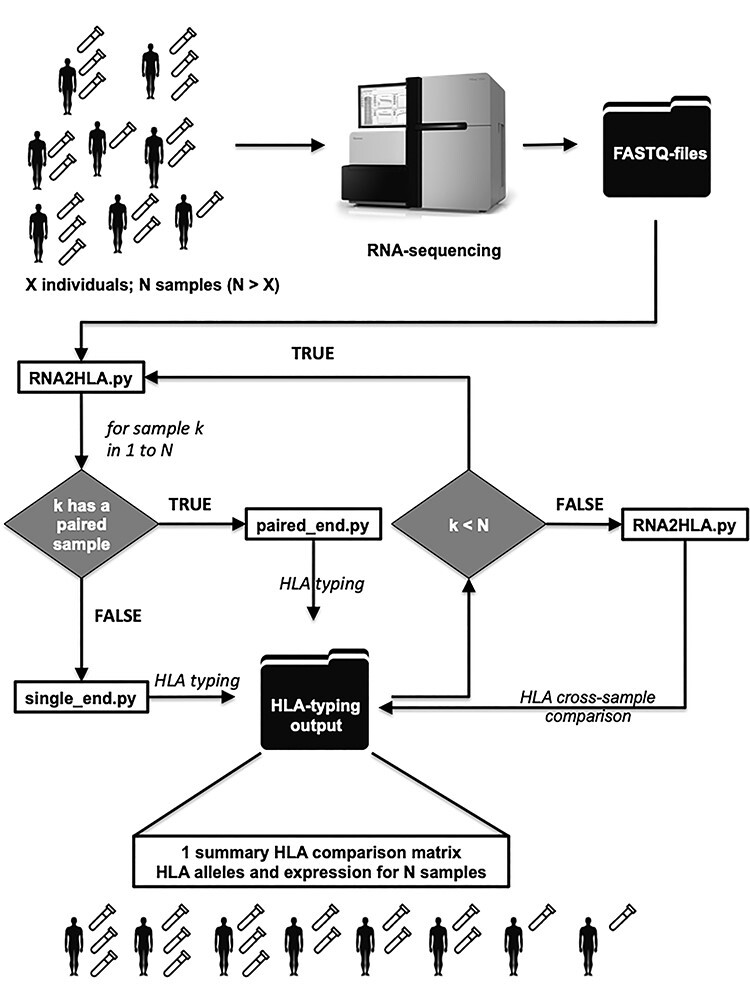
RNA2HLA workflow.

RNA2HLA is a command-line tool, which in the minimal mode does not require anything but a folder containing raw RNA-seq samples in order to produce the study-wise HLA comparison matrix (Figure 1).

The RNA-seq based studies containing *N* (*N* ≥ 1) samples can undergo RNA2HLA analysis, while it serves as a particularly important step of initial QC in the case of *X* individuals included in the study, where *N* > *X*, meaning there are multiple samples related to the same source. Raw RNA-seq data obtained from the sequencing machine used as an input for RNA2HLA—the only mandatory parameter for running the tool is the folder containing raw fastq files, which can be optionally compressed.

Then, each of the samples is extracted and processed sequentially by the relevant HLA-typing subscript, depends on the sample-type (single-end or paired-end), which is accessed independently for each sample within the input folder. The tool extracts the HLA types of the HLA genes of I and II classes, using seq2HLA typing algorithm. Out of six HLA genes (*HLA-A, HLA-B, HLA-C, HLA-DPB1, HLA-DQB1, HLA-DRB1*) with the highest degree of variability, five genes have been selected for RNA2HLA and used for HLA-typing by default, leaving *HLA-DQB1* out, since it has the lowest expression level (Supplementary Figure S1), which decreases the precision of its typing in RNA-seq samples, however, it can be included explicitly by user.

When HLA-typing has been run over all the samples in the input folder, threshold of the *P*-value is applied to all the identified alleles for each sample separately leaving only those alleles, where the confidence of typing passed the significance threshold (user-defined, default *P* < 0.05—suitable for paired-end data; suggested to be moved to *P* < 0.5 for single-end samples). The remaining, confidently typed, HLA alleles are then cross-compared between all of the RNA-seq samples. The percentage and the number of identical alleles for each couple of samples is reported into the squared matrix, along with the total number of alleles successfully identified (Supplementary Table S1).

This summary comparison matrix serves as a main result of the RNA2HLA and can be visualized as a heat map of HLA identities between the samples in the study (e.g. Figure 3, Supplementary Figure S2). Additionally, exact alleles along with corresponding confidence levels and the expression of HLA genes are reported for each sample in an output folder (Supplementary Table S1).

**Figure 2:**
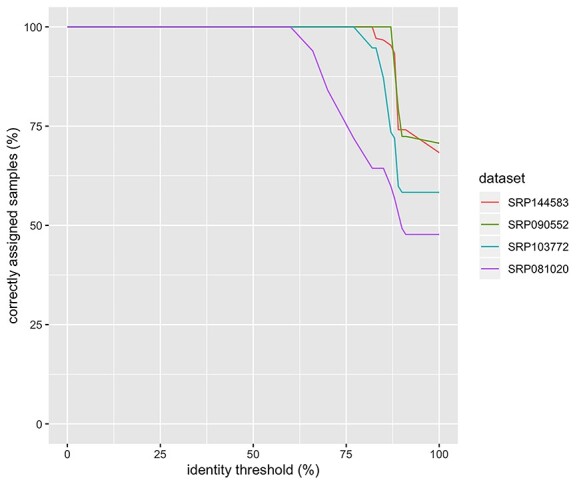
Fraction of correctly assigned samples based on HLA identity threshold for each of the tested studies.

### Dependences

RNA2HLA can be downloaded from Github (https://github.com/Chelysheva/RNA2HLA) [14] and does not require any additional installations. The depository contains one main RNA2HLA Python script along with two subsequent Python scripts (for single-end and paired-end data, respectively), which are called automatically, once the tool is running, and perform the HLA-typing for each sample. The *references* folder contains the databases of the sequences of known HLA alleles of classes I and II to be typed, which were created based on those in the template HLA-typing tool—seq2HLA. Corresponding reference files are selected automatically for the analysis based on the command-line parameter *-g* (see below), which allows choosing between five or six HLA genes. Additional R-scripts contain the relevant functions called during the analysis.

None of the scripts are designed for independent use and are called within the main program setup where relevant. The whole pipeline is completely automated and runs from a single line command in the command-line interface to simplify the processing.

### Required software

RNA2HLA has minimal requirements, thereby allowing a simple and straightforward setup. The tool is written on Python and compatible with both versions—Python 2 and Python 3. The template HLA-typing algorithm seq2HLA was based on Python 2 and was able to perform the typing on paired-end RNA-seq data. For our tool, it has been rewritten on Python 3 and adapted to also allow single-end RNA-seq datasets as an input.

R (version 3.x and higher) has to be installed in order for the subsequent scripts written in R to be functional within the RNA2HLA pipeline.

Bowtie (version 1.1.2 or higher) [15] is used for aligning the RNA-seq reads to the reference files containing the HLA sequences; therefore, it must be installed and reachable from the command-line.

For the ease of the setup, the complete conda [16] environment has been created and can be downloaded (RNA2HLA_env.yml) from the home page of the project at Github.

### Usage and parameters

RNA2HLA takes advantage of a straightforward command-line interface, allowing the whole pipeline to be run from one line of code.

While the minimal required input information is a folder containing raw RNA-seq samples from a study, the additional user-defined parameters are introduced for the advanced users.

The significance threshold for the confidence of HLA-typing can be changed (*-c* parameter), which is particularly relevant for the low-depth datasets, where there are not enough reads to perform the highly confident HLA-typing. Also, in the case of single-end datasets, based on the performances of the typing algorithm (see below), the recommended threshold is *P* < 0.5, which does not, however, decrease the number of correctly assigned alleles, but rather allows including more of them into the final cross-comparison between the samples. All of the RNA-seq datasets used for the benchmarking (Table 2) had a widely acceptable read depth of over 20 million reads per sample, which was enough for confident identification HLA alleles using the default suggested *P*-values of RNA2HLA (<0.05 for paired-end and <0.5 for single-end datasets).

If the analysis is run on a powerful machine with multiple cores, the most time-consuming part of the algorithm—mapping to the corresponding reference HLA sequences with bowtie—can be parallelized by launching a specified number of parallel threads [15]. Each of them runs on a separate core and thereafter significantly speeds-up the whole processing. Including the *-p* parameter allows to define a number of cores used by bowtie.

Another parameter, which can be adjusted, is a number of HLA genes used for the typing. By default, five out of six HLA genes with the highest number of known allele variations are included. The selection was based on the samples used to benchmark the tool: these five genes had higher expression levels and therefore higher confidence of HLA alleles to be correctly identified. Parameter *-g* allows switching between five and six genes included for typing by adding *DQB1* into the algorithm. This is relevant for RNA-seq datasets of a high depth, where the number of reads for the lowly expressed *DQB1* gene is expected to be enough in order to correctly assign HLA alleles with a defined confidence level.

**Table 2:** RNA2HLA performance on available RNA-sequencing datasets. Identity threshold has been defined by the maximum F_1_ score

Dataset	SRP144583	SRP090552	SRP103772	SRP081020
Length (bp)	75	100	51	101
Type	Paired	Paired	Single	Single
# of samples	195	42	53	55
*P*-value	0.05	0.05	0.5	0.5
Identity threshold (%)	81.8	87	77	70
Precision	1	1	0.95	0.99
Recall	1	1	1	0.85
F_1_ score	1	1	0.97	0.91

Abbreviations: Type: paired – paired-end; single – single-end.

### Evaluation of the performance

Two distinct approaches have been used in order to evaluate the performance of RNA2HLA as a QC tool for RNA-seq datasets:

(1) Four publicly available RNA-seq datasets fitting the criteria (containing multiple samples per study participant) were uploaded from Sequence Read Archive (https://www.ncbi.nlm.nih.gov/sra) [17].

(2) RNA-seq datasets were created using simulation, accounting to the HLA frequencies reported in PyPop database (http://pypop.org/popdata/) [18, 19] for each of the selected populations separately.

Among almost 500 of populations included in the database only 12 (Canoncito, Cape_York, Czech, Finn, Japanese, Kimberley, Mixe, Mixteco, Nu, Shona, Zapotec, Zulu) contained the information about the allele frequencies for all of the six HLA genes, which were selected for typing within RNA2HLA (Supplementary Table S2).

RNA-seq datasets for each population has been simulated separately, serving as the strictest evaluation, considering the limited number of alleles present within each reported population. Since none of the studies extended 1000 participants, all the additional alleles from the reference sequences files, which have not been present in the given population, were included with a frequency of 0.001. All the remaining frequencies were adjusted accordingly in order to keep the sum equal 1.

The alleles for each HLA gene have been assigned to each sample randomly accounting for the probabilities described above, using the wgsim simulation tool to simulate RNA-seq samples (https://github.com/lh3/wgsim) [20]. The expression levels were specified based on the estimation of 50 000—100 000 reads mapped to the six selected HLA genes in total—in line with the publicly available RNA-seq studies of medium sequencing depth. The random number of paired-end samples was created assuming the number of participants *X* = 10 and the number of samples per participant 0 < *n* < *X*. Two different read lengths (75 and 100 bp) have been used resulting in creation of two independent RNA-seq datasets per population.

## RESULTS AND DISCUSSION

### Precise HLA-typing of RNA-seq datasets with RNA2HLA

RNA2HLA has been run as a QC tool over four publicly available RNA-seq datasets (SRA: SRP144583 + SRP276081, SRP090552, SRP103772, SRP081020), both single- and paired-end. The number of participants included in the study varied between 42 and 195, while all of the studies had multiple RNA-seq samples taken from the same participant. The read length varied between 51 and 101 bp. RNA2HLA performance was independent of the number of samples or read length (Table 2), yielding both, precision and recall of correct sample pairings across the study, close to 1. Allele identity threshold was slightly higher for paired-end datasets (over 80%) compared to the single-end ones (over 70%), denoting more precise identification of HLA alleles in paired-end RNA-seq data. This trend was expected, as the main advantage of paired-end RNA-seq approach is an increased quality of alignment of the reads, especially in highly variable or repetitive regions of the genome.

RNA2HLA uses seq2HLA typing approach as a template and therefore keeps the precision of the typing in line with original estimations for this tool [9]. This expands the utility of RNA2HLA from being a QC tool—it can also serve as an independent and convenient HLA-typing tool, which takes an advantage of typing the whole RNA-seq based study in one command. The introduction of such option increases the value of RNA2HLA, since it has not been possible to perform a study-wide HLA-typing with any of the previously developed tools. All of the HLA-typing tools had to be run separately for each of the samples within the study to produce independent outputs without cross-comparison and summarization, which are crucial for in the case of extensive sample-size.

For the evaluation of the RNA2HLA performance, the definition of the identity threshold was based on maximization of F_1_ score—harmonic mean of the precision and recall values, which is a widely used measure to assess the computational approaches, including omics tools [21]. For majority of the studies, 100% individuals can be correctly assigned using HLA identity threshold over 80% between the samples for paired-end, or over 70% for single-end data (Table 1, Figure 2). We propose these thresholds to be used as default cut-offs for the samples to be considered from the same source while reading the output of RNA2HLA.

**Figure 3:**
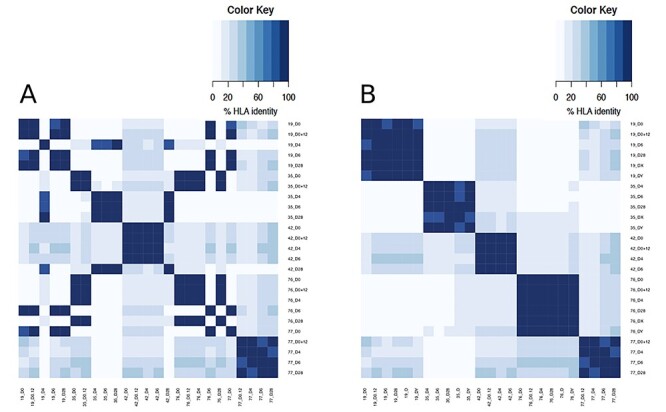
Heat maps of HLA identity: before (A) and after (B) the correction of sample labeling.

In the RNA2HLA application by typical user, the threshold does not require a complex calculation, but should rather be intuitive, since the samples, which do not relate to one individual, almost never have over 50% identical HLA alleles and can be clearly distinguished, while any mismatched samples would be outstanding (Figure 3).

### RNA2HLA reveals mislabeled samples within publicly available RNA-seq datasets

Strikingly, besides correctly grouping the samples from the same individual based on their HLA-identity, RNA2HLA was able to confidently reveal the wrongly assigned (e.g. mislabeled) samples between the individuals within 2 (SRP144583 + SRP276081 and SRP081020) out of four studies used for the benchmarking. Importantly, all of these datasets were already deposited online were previously published [22, 23, 24, 25] including those with identified mislabeling [24, 25]. This finding highlights the crucial importance of RNA2HLA to be introduced as a QC tool for the RNA-seq based studies.

The first study, where the mislabeling has been identified, was a paired-end RNA-seq dataset (SRP144583 + SRP276081, read length – 75 bp). The study analyzed the transcriptional responses in whole blood of 80 healthy adults, which were experimentally challenged with *Salmonella enterica* serovar Paratyphi A. Single oral dose of *S*. Paratyphi was given in two dose levels: high (HD) and low (LD), the RNA-seq samples have been collected at various time points (D0, D0 + 12 h, D4, D6, D28).

RNA2HLA identified multiple mislabeled samples (6 in total), which were assigned to the wrong study participants. This dataset has been partially included in the publication [24] involving two of the misallocated samples (SRR7119070, SRR7119072). These RNA-seq samples were used to reveal diagnostic gene signature, which would allow distinguishing enteric fever from other febrile diseases.

One of the mislabeled samples (SRR7119072, in the study – P1_77_D0) has been misclassified by the diagnostic prediction signature [24], while the corrected allocation of the sample would have been improved the performance of the predicting algorithm.

RNA2HLA was able to correctly reassign all of the mismatched samples to the right individuals (Figure 3, Supplementary Table S3—before and after correction; only mismatched sample groups are included). However, in this particular study, the time point, when the sample was taken (D0, D0 + 12 h, D4, D6 or D28), carried crucial information about the transcriptomic responses, which were relevant for the analysis. Therefore, once the mislabeling has been identified, all of the mislabeled samples would have to be excluded from the follow-up processing and further analysis of this dataset.

The second mislabeled dataset (SRP081020, single-end RNA-seq, read length – 101 bp), did not have a corresponding publication from the supplier at the moment when RNA2HLA analysis has been performed, however, the initial analysis and normalization were done and publically available. This study used RNA-seq dataset to profile gene expression of the host immune response to an irradiated sporozoite immunization (PvRAS) and subsequent *Plasmodium vivax* malaria challenge. RNA-seq samples have been collected from 20 participants of three different study groups (3 Control, 5 Duffy Fy(−) and 12 PvRAS immunized) at three time points—diagnosis day, baseline and pre-challenge day.

Our HLA-based QC tool was able to identify a sample (SRR4005716, time point – diagnosis), which HLA-identity did not fit to the corresponding sample labeling (Supplementary Table S4). Strikingly, once the mislabeled sample was reassigned based on HLA types, it corresponded to another study group, where the participant was susceptible to *P. vivax* infection instead of being protected. However, there was already another sample taken from the same individual at time point – diagnosis (SRR4005709). It remains unclear, at which time point this mislabeled sample has been collected, since all of the time points were already present for this participant (SRR4005693, SRR4005709 and SRR4005728). Moreover, the correction of this sample led to the situation when one of the 20 participants of the study had only one sample left in the dataset (SRR4005735 – collected at pre-challenge day), which made it impractical for the downstream analysis of this dataset.

This study has been previously analyzed and included in the publication from another group among multiple publicly available datasets [25]. Authors identified the mislabeled sample (SRR4005716) to be uninfected, while the actual reason for that was not the protection from *P. vivax*, but rather the mislabeling and the wrongly assigned time point. Taking into account the correction, this sample should have been excluded from their analysis.

Running RNA2HLA on these two datasets (SRP144583 + SRP276081 and SRP081020) demonstrated the successful identification of multiple events of the misallocation of the samples—assigning them to the wrong individuals and thereafter to the wrong study groups—this mistake could have a significant effect on the outcome of the research project.

These findings confirm that using HLA-typing as a QC method can improve labeling accuracy and therefore downstream bioinformatics analysis.

### RNA2HLA benchmarking using strict population-based simulated RNA-seq datasets

Another, more restricted step of evaluation of RNA2HLA performance, was based on the simulated RNA-seq datasets, which were created using available HLA-allele frequencies from 12 various populations. As the sample size for each of the simulated RNA-seq studies has been random, fraction of correctly assigned samples at a given HLA identity threshold was chosen as criteria for the evaluation. In line with the real RNA-seq datasets described above, even in this unnaturally restricted case, the majority of the samples have been successfully assigned to the correct individuals at the identity threshold over 75% (Supplementary Table S5, Supplementary Figure S2) for each of the simulated RNA-seq datasets.

Only two populations (Mixe, Kimberley) with a lowest number of total known alleles for all of the six HLA genes (47 and 41, respectively) revealed the decreased precision of HLA-typing, which led to the problems of the correct assignment of the samples within these two datasets. Therefore, the total number of alleles, which were present in the particular population, played a crucial role for the correct allocation, low HLA variability within the frequency dataset led to the higher amount of similar alleles and homozygous samples to be present within the simulated RNA-seq data. However, the dataset from another population with low allele variability—Canoncito (46 HLA alleles in total) has been assigned correctly, which can be potentially explained by the presence of more distant HLA alleles, which had less sequence similarity, within this population. Taking into account, that the population studies had a limited number of participants, lower than 1000 each, the allele distribution (even adjusted to the ‘potential’ rare alleles, which were missing in the initial study), was far more strict compared to the presence of the alleles in the real populations. Therefore, this limitation, observed within the simulated RNA-seq datasets, does not cause any problems in vast majority of the datasets in the natural study environment.

## LIMITATIONS

In the case of studying a particular population with prior knowledge of the low HLA allele diversity—in analog with two of the simulated datasets above—RNA2HLA should not be used as a QC, but only as a convenient study-wide HLA-typing method. One can refer to the Allele Frequency Net Database [26], which contains the information of the populations (over 4200 studies), which can be accessed through the interactive HLA world map. The populations with less than 50 of total known alleles should be considered as of low diversity.

Another point, which may cause a potential limitation to the QC component of RNA2HLA, is the existence of linkage disequilibrium (LD)—non-random association of alleles at different loci in a given population. Even so, LD has been reported for HLA genes [27], there is limited knowledge of the exact LD and it is stretch in the majority of the populations and generally should not affect the analysis, as we have been able to show by successfully performing the analysis in all of example studies. If the known LD exists in the dataset, the QC part of RNA2HLA should be used with caution, and in the further versions of the tool (>1.0) should be adapted by including only those HLA genes, which are not in LD (see the Future development -*g* option).

## FUTURE DEVELOPMENT

Gene expression levels may vary across the datasets and input sources, therefore, any of the potential combinations of HLA genes should be used for RNA2HLA comparison. The exact user-defined gene list will be included in the selection (*-g* option).

We have shown that heat maps serve as the best visualization of HLA comparison matrix, so this option will be automated and included in the updated version of the program.

Automatic calculation of the diversity of the HLA alleles in the study based on the total number of samples (and sources – can be included as additional option *-s*) will allow the users to be confident about QC without consulting the allele frequency net database, or, in the case of the low diversity being detected in the dataset, the user will receive a warning message.

RNA2HLA is a first tool allowing HLA-typing on single-end RNA-seq datasets with a reasonable precision; however, algorithm can be further improved and adjusted to improve the performance on single-end data, including the appropriate adjustment of *P*-value calculation, which is currently performed similarly to paired-end data.

**Key Points**

- RNA2HLA – a novel command-line tool, which serves as a QC for the RNA-seq studies allowing precise allocation and grouping of RNA samples based on their HLA types.
- RNA2HLA revealed multiple events of mislabeling within publicly available datasets—showing this being not an infrequent event in RNA-seq studies.
- RNA2HLA is an important tool for improving the bioinformatics processing pipelines for RNA-seq datasets that contain multiple samples from the common source/participant.
- The tool successfully extracts HLA alleles and can be used to perform massive HLA-typing from RNA-seq study even if matching of the samples is not required.

## DATA AVAILABILITY

The datasets analyzed during the current study are available in the Sequence Read Archive (https://www.ncbi.nlm.nih.gov/sra/) under the following accession numbers: SRP103772, SRP081020, SRP090552, SRP144583, SRP276081.

## SOFTWARE AVAILABILITY AND REQUIREMENTS

Project name: RNA2HLA

Project home page: https://github.com/Chelysheva/RNA2HLA

Operating system(s): Platform independent

Programming language: Python (2 or 3), R (3.x or higher, developed with 3.6.3)

Other requirements: Bowtie (developed with 1.1.2), biopython (Python2-1.76, Python3-1.76 or higher), numpy (Python2-1.16-1.18, Python3-1.16 or higher), pandas (Python2-0.24.2, Python3-0.24.2 or higher)

Complete conda environment: Available at Github (RNA2HLA_env.yml)

## LIST OF ABBREVIATIONS

RNA-seq: RNA-sequencing; MHC: major histocompatibility complex; HLA: human leucocyte antigen; NGS: next generation sequencing; QC: quality control; SNP: single nucleotide polymorphism; bp: base pairs; OS - operating system; LD: linkage disequilibrium.

## AUTHORS’ CONTRIBUTIONS

I.C. designed and programmed the RNA2HLA, processed and analyzed the data, prepared figures and wrote the manuscript. A.J.P. made contributions to the conception and design of the study and revised the manuscript. D.O’C. conceived the project, supervised the study and edited the manuscript. All authors reviewed and approved the manuscript.

## Supplementary Material

Suppl_figure_1_bbab055

Suppl_figure_2_bbab055

Suppl_table_1_bbab055

Suppl_table_2_bbab055

Suppl_table_3_bbab055

Suppl_table_4_bbab055

Suppl_table_5_bbab055

## Notes

**Irina Chelysheva**, PhD, is a postdoctoral medical bioinformatician at the Oxford Vaccine Group, she works on the analysis of various NGS datasets (including RNA-seq, ONT-seq, Ribo-seq and TCR-seq) for over 6 years.

**Andrew J Pollard** is the Director of the Oxford Vaccine Group, Professor of paediatric infection and immunity at the University of Oxford, Honorary Consultant Paediatrician at Oxford Children’s Hospital.

**Daniel O’Connor** DPhil, is a postdoctoral researcher at the Oxford Vaccine Group, he has both a wet-laboratory and bioinformatics background, his main research interests relate to the analysis of contemporary, high-dimensional datasets to elucidate the molecular determinants of vaccine responses.
