# Supplementary figures and images for: RNA2HLA: HLA-based quality control of RNA-seq datasets
Source: Brief Bioinform. 2021 Mar 24;22(5):bbab055. doi: 10.1093/bib/bbab055 (PMC8425422; doi:10.1093/bib/bbab055)

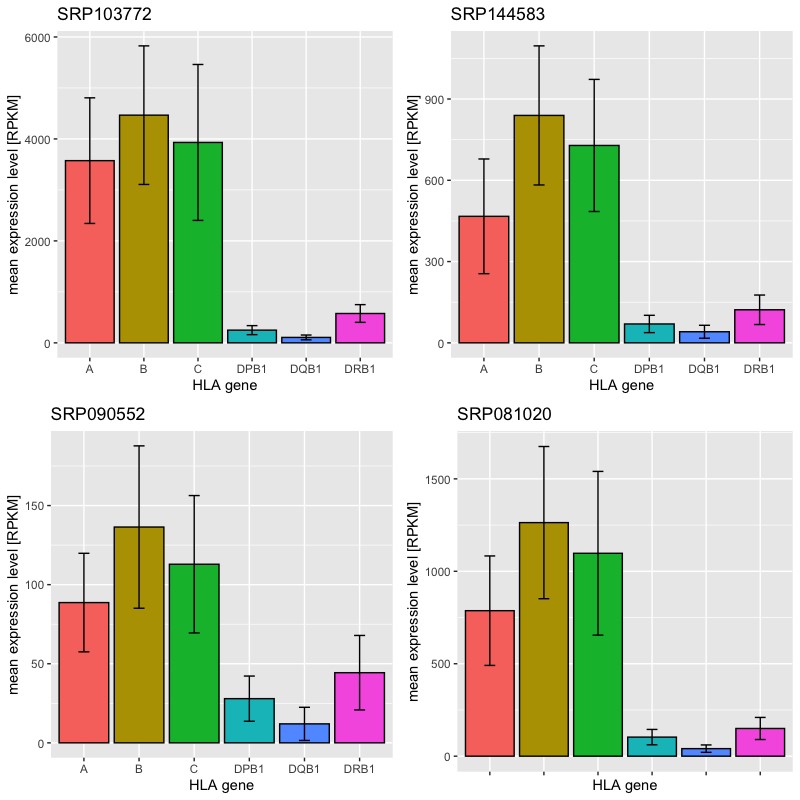

Supplement: Suppl_figure_1_bbab055 [file suppl_figure_1_bbab055.jpeg]
