# Supplementary material for: RNA2HLA: HLA-based quality control of RNA-seq datasets
Source: Brief Bioinform. 2021 Mar 24;22(5):bbab055. doi: 10.1093/bib/bbab055 (PMC8425422; doi:10.1093/bib/bbab055)
Supplement: Suppl_figure_2_bbab055 [file suppl_figure_2_bbab055.pdf]

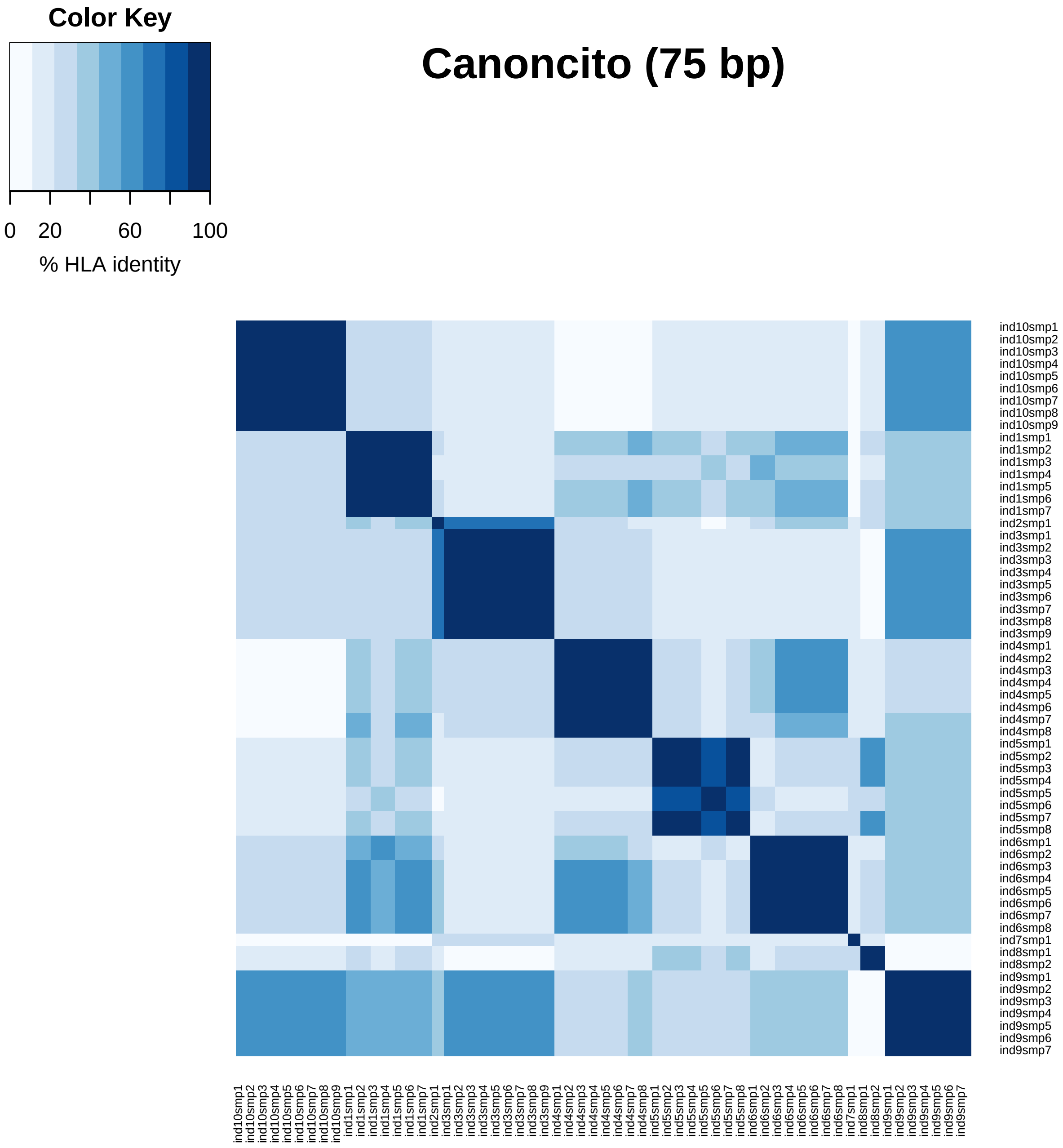
Color Key
0
20
60
100
% HLA identity
Canoncito (75 bp)
ind10smp1
ind10smp2
ind10smp3
ind10smp4
ind10smp5
ind10smp6
ind10smp7
ind10smp8
ind10smp9
ind1smp1
ind1smp2
ind1smp3
ind1smp4
ind1smp5
ind1smp6
ind1smp7
ind2smp1
ind3smp1
ind3smp2
ind3smp3
ind3smp4
ind3smp5
ind3smp6
ind3smp7
ind3smp8
ind3smp9
ind4smp1
ind4smp2
ind4smp3
ind4smp4
ind4smp5
ind4smp6
ind4smp7
ind4smp8
ind5smp1
ind5smp2
ind5smp3
ind5smp4
ind5smp5
ind5smp6
ind5smp7
ind5smp8
ind6smp1
ind6smp2
ind6smp3
ind6smp4
ind6smp5
ind6smp6
ind6smp7
ind6smp8
ind7smp1
ind8smp1
ind8smp2
ind9smp1
ind9smp2
ind9smp3
ind9smp4
ind9smp5
ind9smp6
ind9smp7

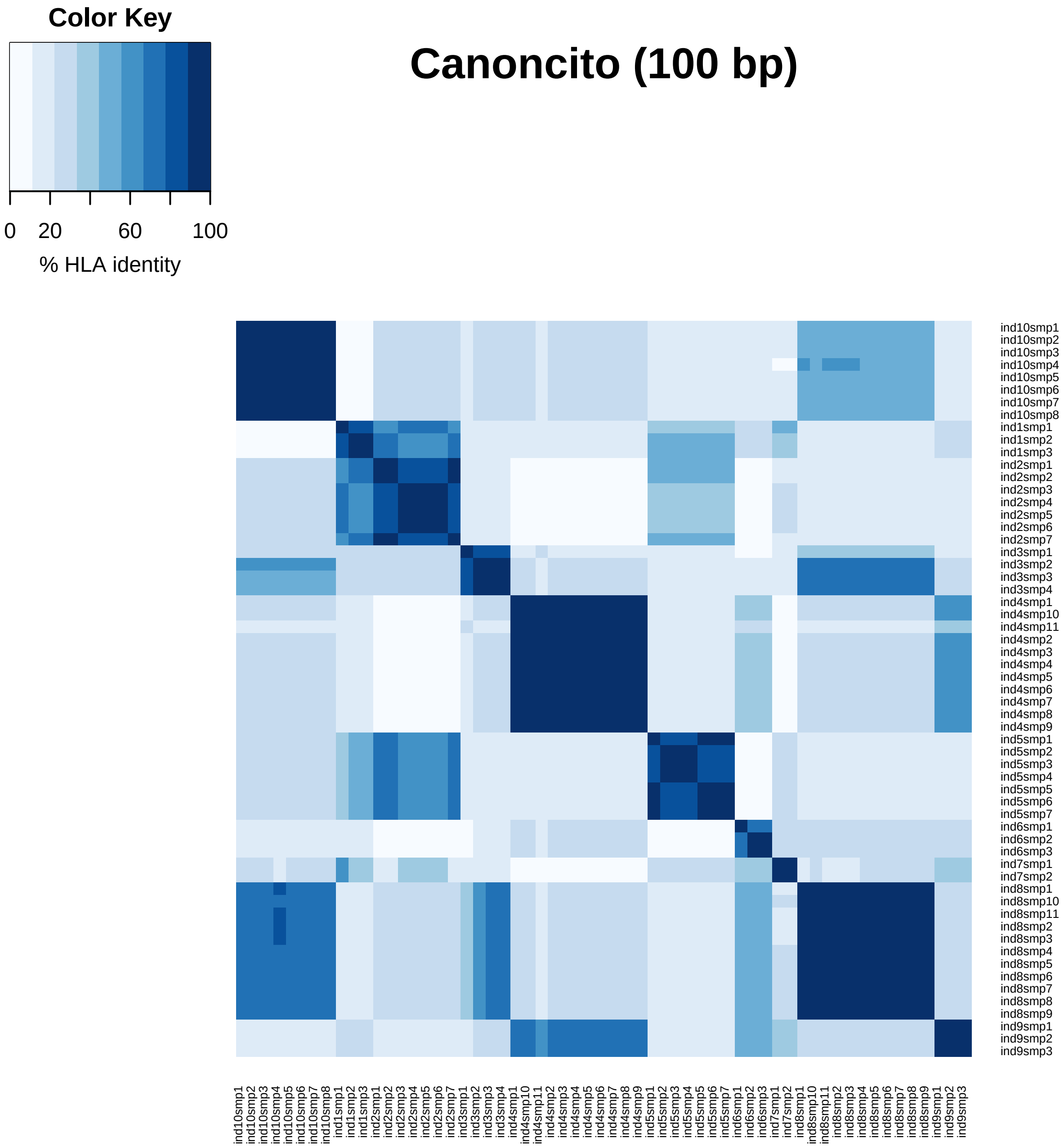

Color Key
0
20
60
100
% HLA identity
Canoncito (100 bp)
ind10smp1
ind10smp2
ind10smp3
ind10smp4
ind10smp5
ind10smp6
ind10smp7
ind10smp8
ind1smp1
ind1smp2
ind1smp3
ind2smp1
ind2smp2
ind2smp3
ind2smp4
ind2smp5
ind2smp6
ind2smp7
ind3smp1
ind3smp2
ind3smp3
ind3smp4
ind4smp1
ind4smp10
ind4smp11
ind4smp2
ind4smp3
ind4smp4
ind4smp5
ind4smp6
ind4smp7
ind4smp8
ind4smp9
ind5smp1
ind5smp2
ind5smp3
ind5smp4
ind5smp5
ind5smp6
ind5smp7
ind6smp1
ind6smp2
ind6smp3
ind7smp1
ind7smp2
ind8smp1
ind8smp10
ind8smp11
ind8smp2
ind8smp3
ind8smp4
ind8smp5
ind8smp6
ind8smp7
ind8smp8
ind8smp9
ind9smp1
ind9smp2
ind9smp3
ind10smp1
ind10smp2
ind10smp3
ind10smp4
ind10smp5
ind10smp6
ind10smp7
ind10smp8
ind1smp1
ind1smp2
ind1smp3
ind2smp1
ind2smp2
ind2smp3
ind2smp4
ind2smp5
ind2smp6
ind2smp7
ind3smp1
ind3smp2
ind3smp3
ind3smp4
ind4smp1
ind4smp10
ind4smp11
ind4smp2
ind4smp3
ind4smp4
ind4smp5
ind4smp6
ind4smp7
ind4smp8
ind4smp9
ind5smp1
ind5smp2
ind5smp3
ind5smp4
ind5smp5
ind5smp6
ind5smp7
ind6smp1
ind6smp2
ind6smp3
ind7smp1
ind7smp2
ind8smp1
ind8smp10
ind8smp11
ind8smp2
ind8smp3
ind8smp4
ind8smp5
ind8smp6
ind8smp7
ind8smp8
ind8smp9
ind9smp1
ind9smp2
ind9smp3

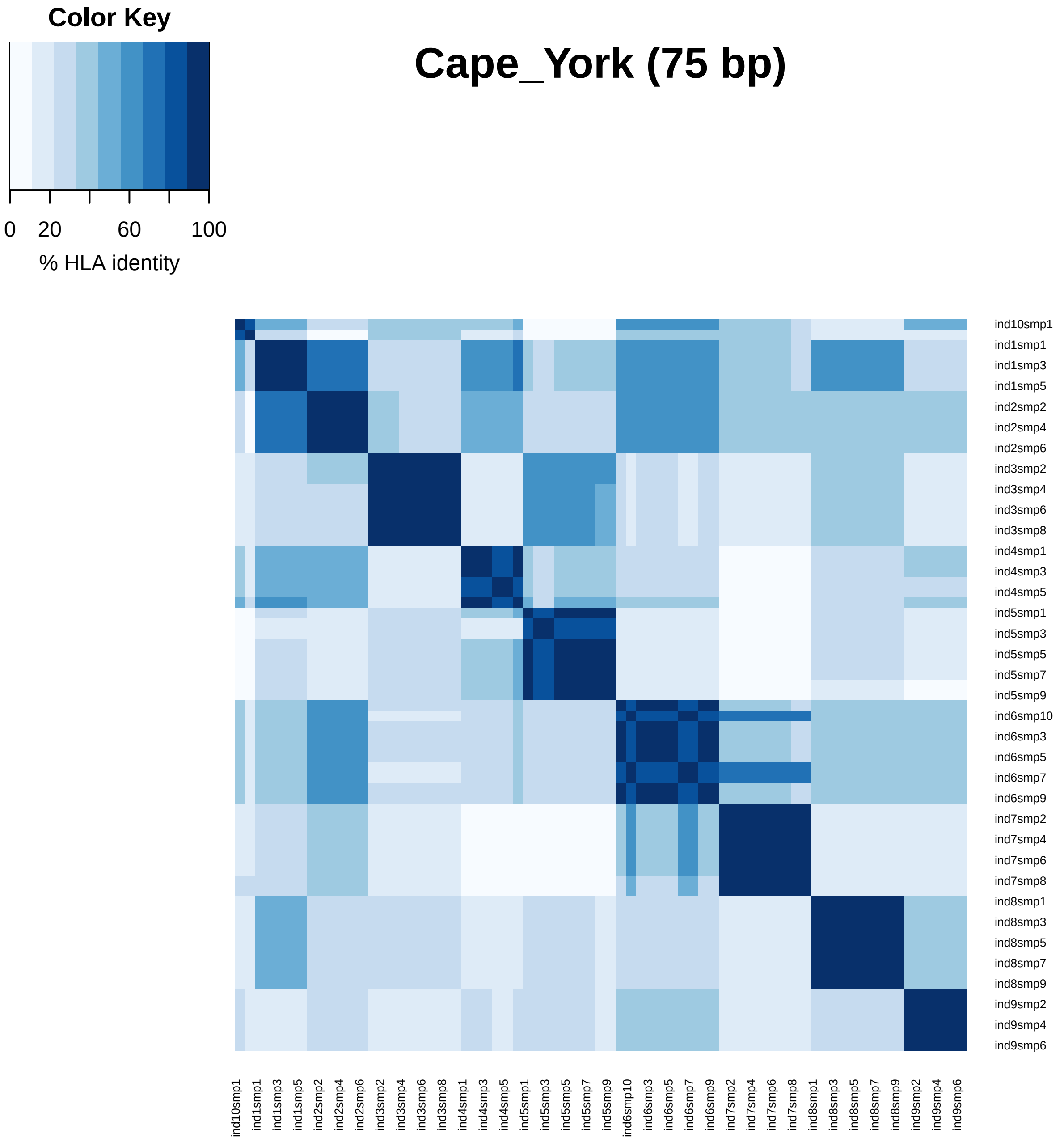
Color Key
0
20
60
100
% HLA identity
Cape_York (75 bp)
ind10smp1
ind1smp1
ind1smp3
ind1smp5
ind2smp2
ind2smp4
ind2smp6
ind3smp2
ind3smp4
ind3smp6
ind3smp8
ind4smp1
ind4smp3
ind4smp5
ind5smp1
ind5smp3
ind5smp5
ind5smp7
ind5smp9
ind6smp10
ind6smp3
ind6smp5
ind6smp7
ind6smp9
ind7smp2
ind7smp4
ind7smp6
ind7smp8
ind8smp1
ind8smp3
ind8smp5
ind8smp7
ind8smp9
ind9smp2
ind9smp4
ind9smp6

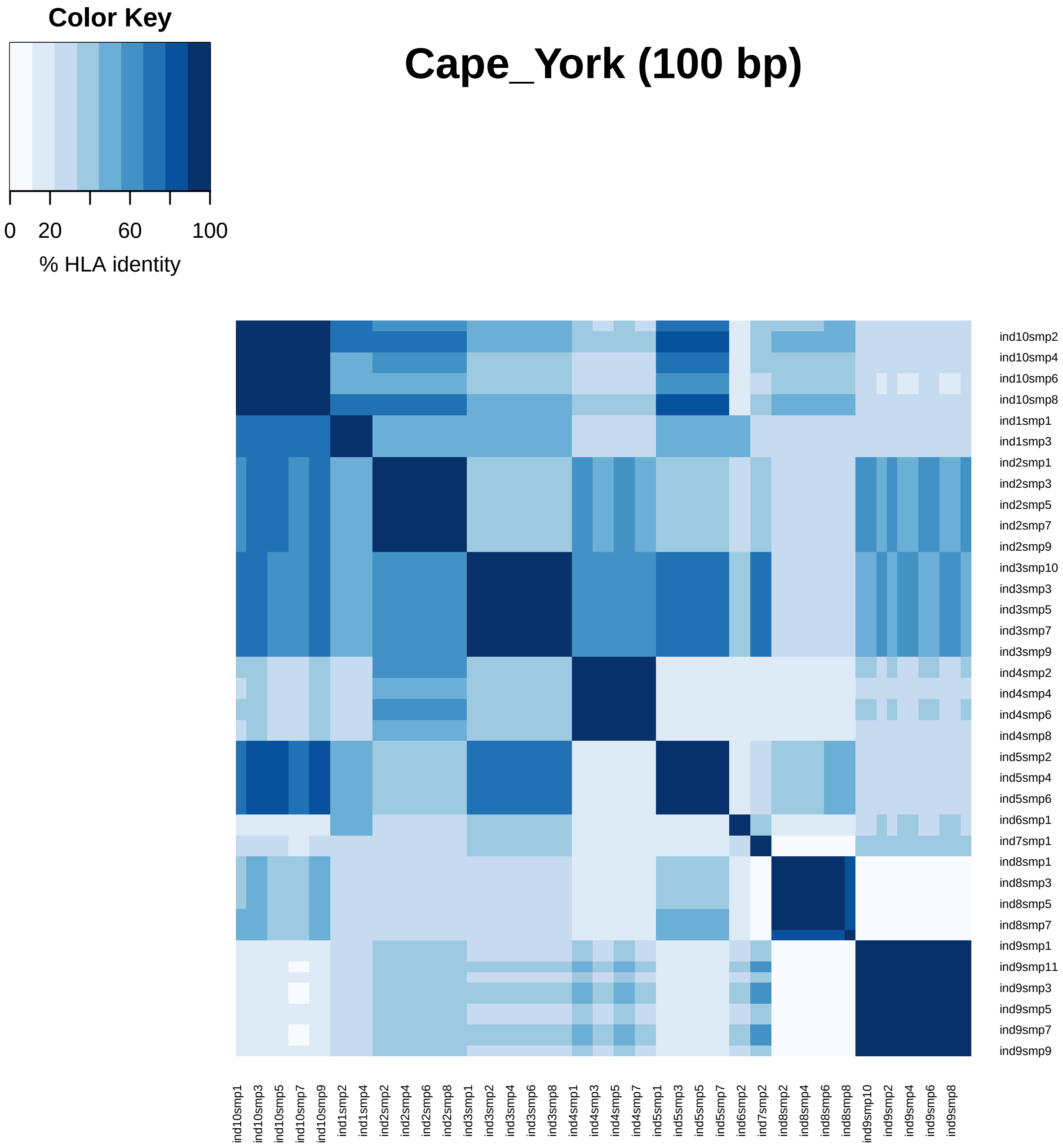
Color Key
0
20
60
100
% HLA identity
Cape_York (100 bp)
ind10smp2
ind10smp4
ind10smp6
ind10smp8
ind1smp1
ind1smp3
ind2smp1
ind2smp3
ind2smp5
ind2smp7
ind2smp9
ind3smp10
ind3smp3
ind3smp5
ind3smp7
ind3smp9
ind4smp2
ind4smp4
ind4smp6
ind4smp8
ind5smp2
ind5smp4
ind5smp6
ind6smp1
ind7smp1
ind8smp1
ind8smp3
ind8smp5
ind8smp7
ind9smp1
ind9smp11
ind9smp3
ind9smp5
ind9smp7
ind9smp9
ind10smp1
ind10smp3
ind10smp5
ind10smp7
ind10smp9
ind1smp2
ind1smp4
ind2smp2
ind2smp4
ind2smp6
ind2smp8
ind3smp1
ind3smp2
ind3smp4
ind3smp6
ind3smp8
ind4smp1
ind4smp3
ind4smp5
ind4smp7
ind5smp1
ind5smp3
ind5smp5
ind5smp7
ind6smp2
ind7smp2
ind8smp2
ind8smp4
ind8smp6
ind8smp8
ind9smp10
ind9smp2
ind9smp4
ind9smp6
ind9smp8

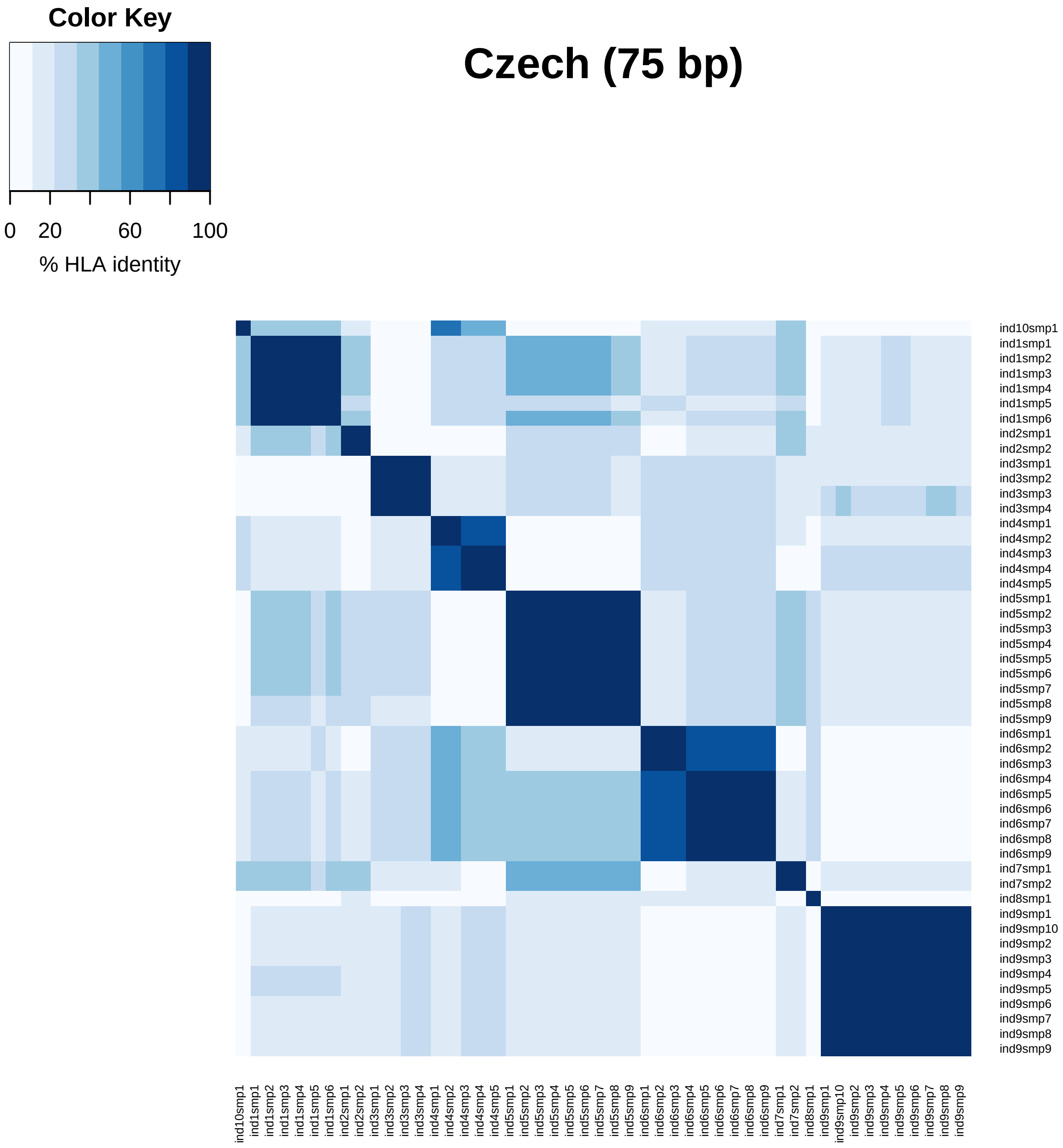

Color Key
0
20
60
100
% HLA identity
Czech (75 bp)
ind10smp1
ind1smp1
ind1smp2
ind1smp3
ind1smp4
ind1smp5
ind1smp6
ind2smp1
ind2smp2
ind3smp1
ind3smp2
ind3smp3
ind3smp4
ind4smp1
ind4smp2
ind4smp3
ind4smp4
ind4smp5
ind5smp1
ind5smp2
ind5smp3
ind5smp4
ind5smp5
ind5smp6
ind5smp7
ind5smp8
ind5smp9
ind6smp1
ind6smp2
ind6smp3
ind6smp4
ind6smp5
ind6smp6
ind6smp7
ind6smp8
ind6smp9
ind7smp1
ind7smp2
ind8smp1
ind9smp1
ind9smp10
ind9smp2
ind9smp3
ind9smp4
ind9smp5
ind9smp6
ind9smp7
ind9smp8
ind9smp9

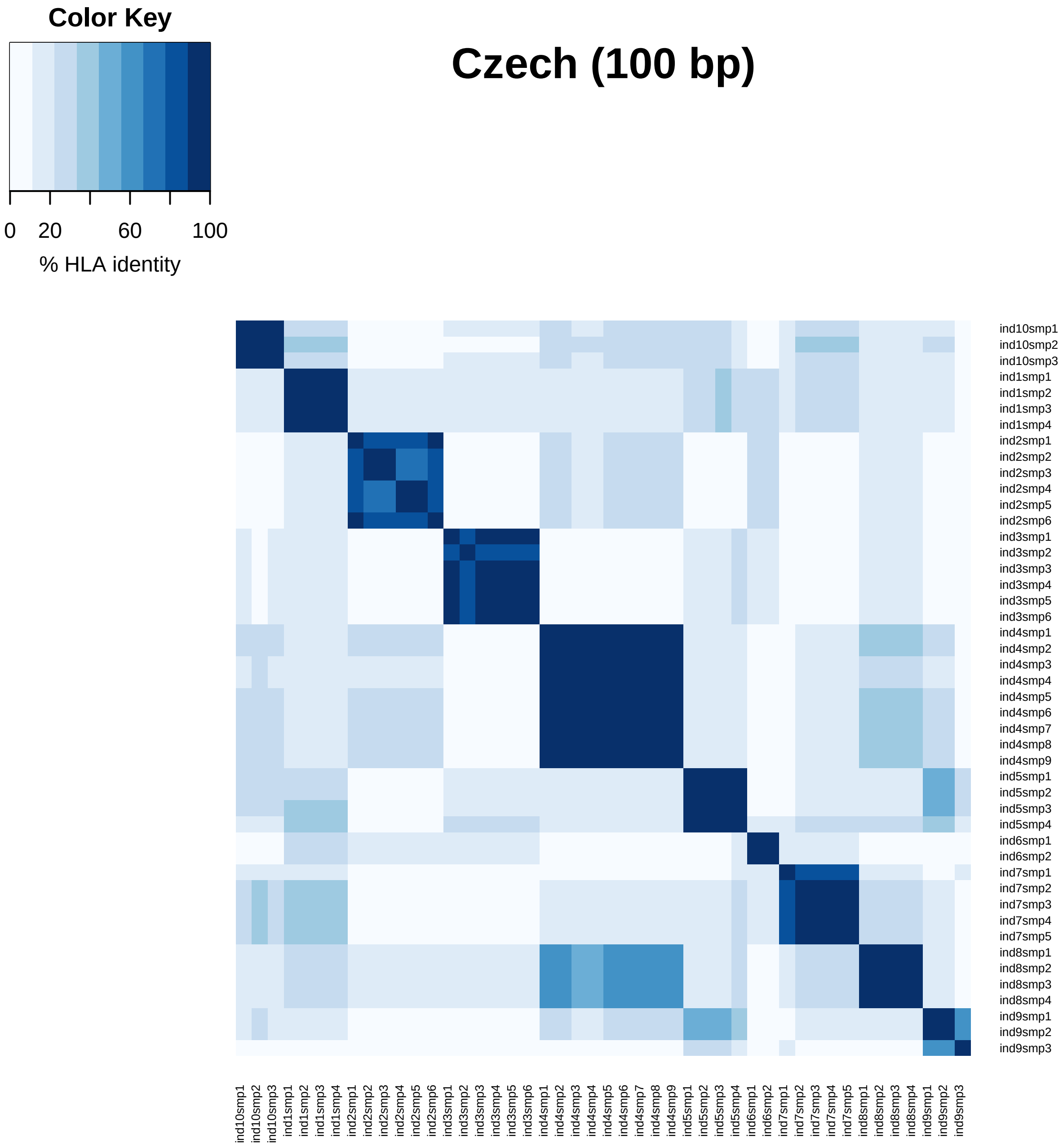
Color Key
0
20
60
100
% HLA identity
Czech (100 bp)
ind10smp1
ind10smp2
ind10smp3
ind1smp1
ind1smp2
ind1smp3
ind1smp4
ind2smp1
ind2smp2
ind2smp3
ind2smp4
ind2smp5
ind2smp6
ind3smp1
ind3smp2
ind3smp3
ind3smp4
ind3smp5
ind3smp6
ind4smp1
ind4smp2
ind4smp3
ind4smp4
ind4smp5
ind4smp6
ind4smp7
ind4smp8
ind4smp9
ind5smp1
ind5smp2
ind5smp3
ind5smp4
ind6smp1
ind6smp2
ind7smp1
ind7smp2
ind7smp3
ind7smp4
ind7smp5
ind8smp1
ind8smp2
ind8smp3
ind8smp4
ind9smp1
ind9smp2
ind9smp3

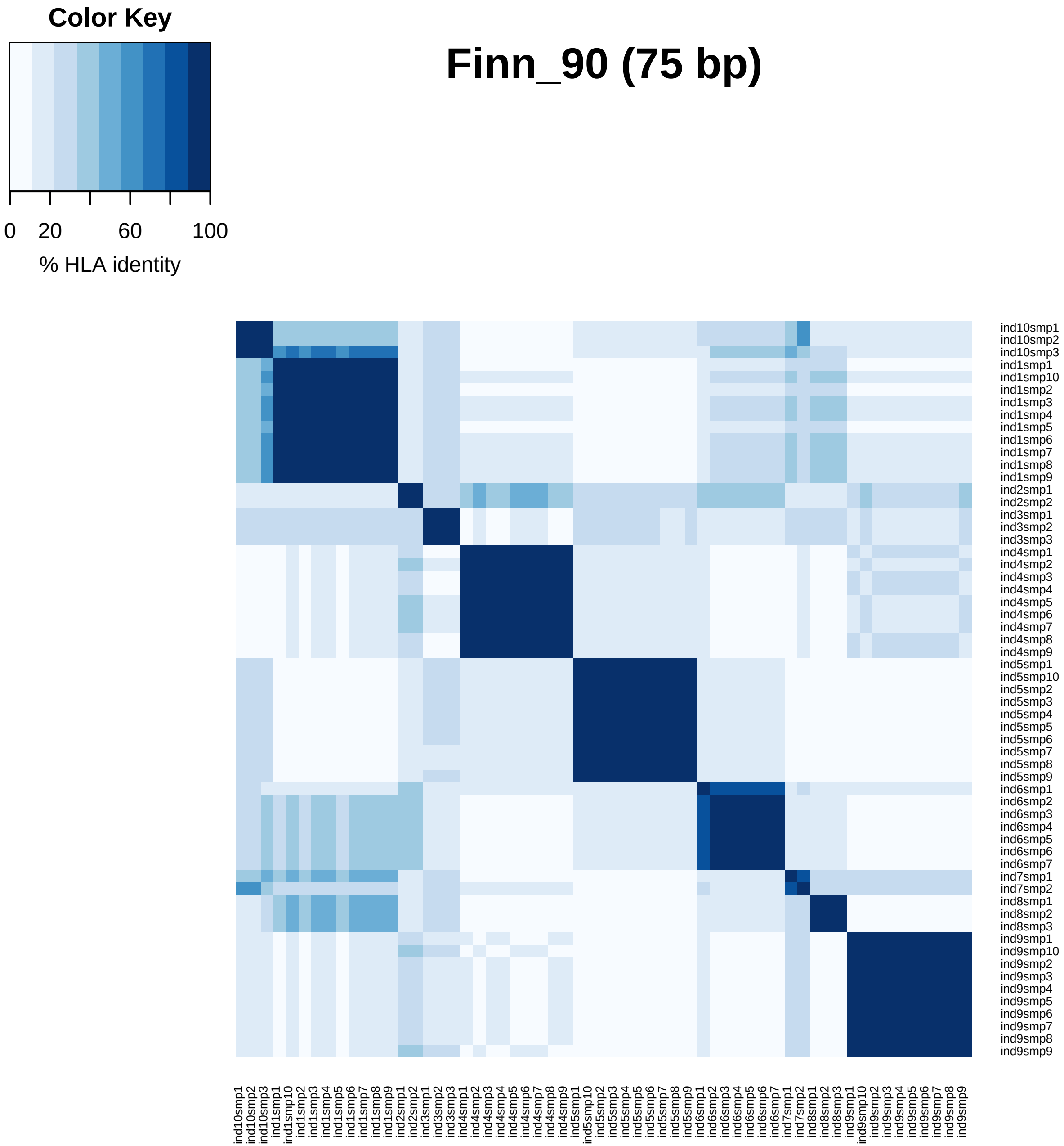
Color Key
0
20
60
100
% HLA identity
Finn_90 (75 bp)
ind10smp1
ind10smp2
ind10smp3
ind1smp1
ind1smp10
ind1smp2
ind1smp3
ind1smp4
ind1smp5
ind1smp6
ind1smp7
ind1smp8
ind1smp9
ind2smp1
ind2smp2
ind3smp1
ind3smp2
ind3smp3
ind4smp1
ind4smp2
ind4smp3
ind4smp4
ind4smp5
ind4smp6
ind4smp7
ind4smp8
ind4smp9
ind5smp1
ind5smp10
ind5smp2
ind5smp3
ind5smp4
ind5smp5
ind5smp6
ind5smp7
ind5smp8
ind5smp9
ind6smp1
ind6smp2
ind6smp3
ind6smp4
ind6smp5
ind6smp6
ind6smp7
ind7smp1
ind7smp2
ind8smp1
ind8smp2
ind8smp3
ind9smp1
ind9smp10
ind9smp2
ind9smp3
ind9smp4
ind9smp5
ind9smp6
ind9smp7
ind9smp8
ind9smp9

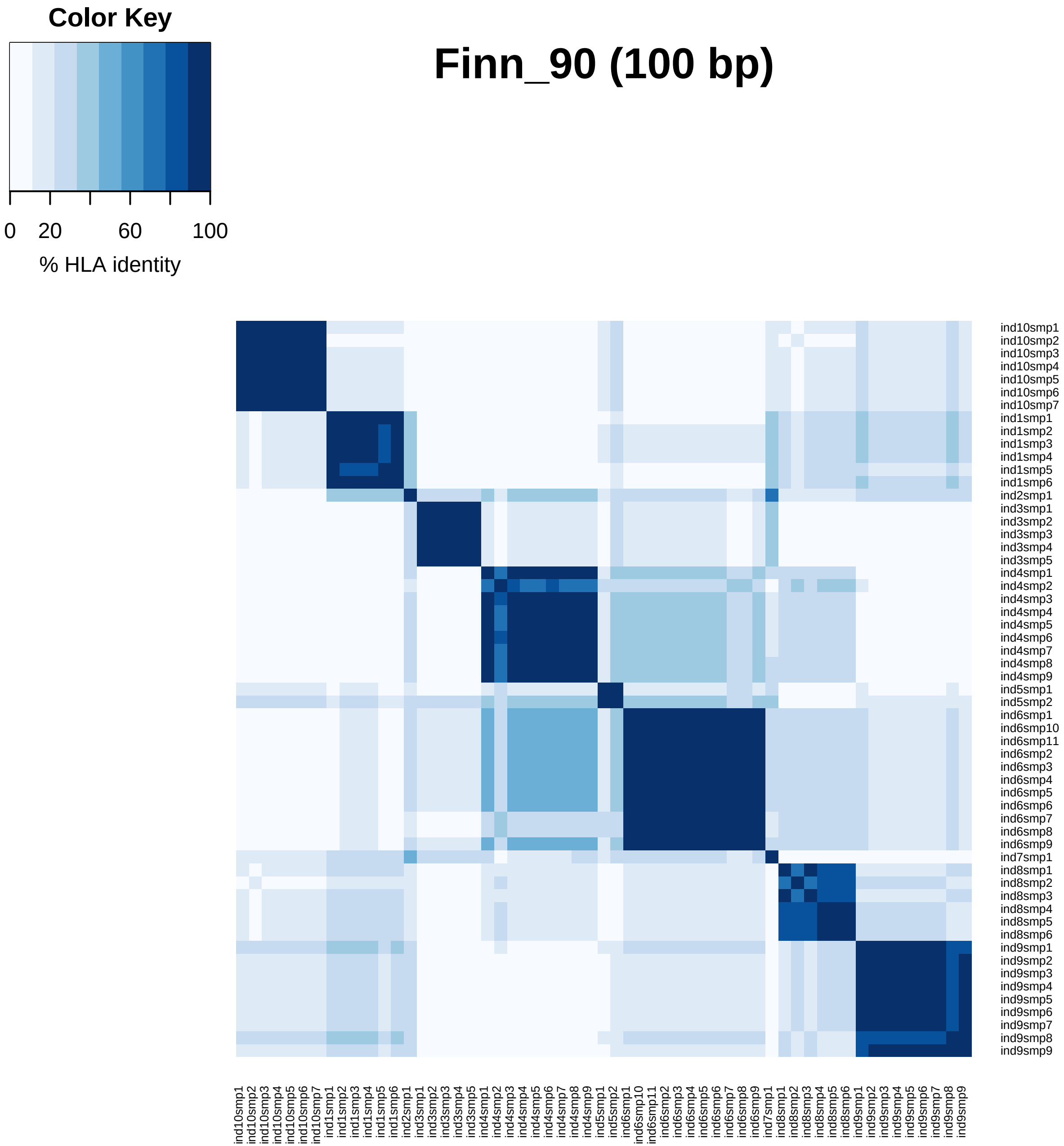
Color Key
0
20
60
100
% HLA identity
Finn_90 (100 bp)
ind10smp1
ind10smp2
ind10smp3
ind10smp4
ind10smp5
ind10smp6
ind10smp7
ind1smp1
ind1smp2
ind1smp3
ind1smp4
ind1smp5
ind1smp6
ind2smp1
ind3smp1
ind3smp2
ind3smp3
ind3smp4
ind3smp5
ind4smp1
ind4smp2
ind4smp3
ind4smp4
ind4smp5
ind4smp6
ind4smp7
ind4smp8
ind4smp9
ind5smp1
ind5smp2
ind6smp1
ind6smp10
ind6smp11
ind6smp2
ind6smp3
ind6smp4
ind6smp5
ind6smp6
ind6smp7
ind6smp8
ind6smp9
ind7smp1
ind8smp1
ind8smp2
ind8smp3
ind8smp4
ind8smp5
ind8smp6
ind9smp1
ind9smp2
ind9smp3
ind9smp4
ind9smp5
ind9smp6
ind9smp7
ind9smp8
ind9smp9

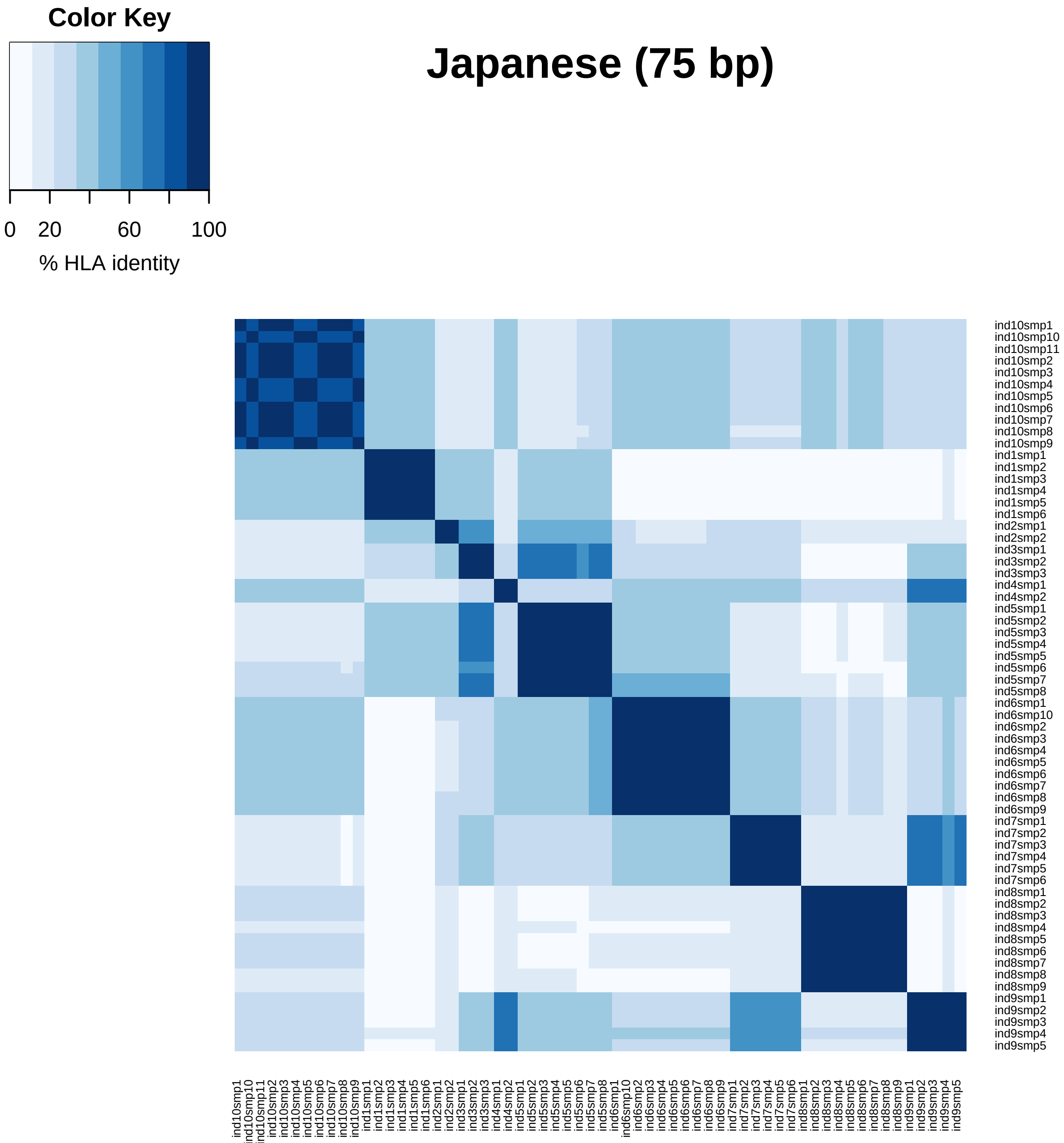

Color Key
0
20
60
100
% HLA identity
Japanese (75 bp)
ind10smp1
ind10smp10
ind10smp11
ind10smp2
ind10smp3
ind10smp4
ind10smp5
ind10smp6
ind10smp7
ind10smp8
ind10smp9
ind1smp1
ind1smp2
ind1smp3
ind1smp4
ind1smp5
ind1smp6
ind2smp1
ind2smp2
ind3smp1
ind3smp2
ind3smp3
ind4smp1
ind4smp2
ind5smp1
ind5smp2
ind5smp3
ind5smp4
ind5smp5
ind5smp6
ind5smp7
ind5smp8
ind6smp1
ind6smp10
ind6smp2
ind6smp3
ind6smp4
ind6smp5
ind6smp6
ind6smp7
ind6smp8
ind6smp9
ind7smp1
ind7smp2
ind7smp3
ind7smp4
ind7smp5
ind7smp6
ind8smp1
ind8smp2
ind8smp3
ind8smp4
ind8smp5
ind8smp6
ind8smp7
ind8smp8
ind8smp9
ind9smp1
ind9smp2
ind9smp3
ind9smp4
ind9smp5

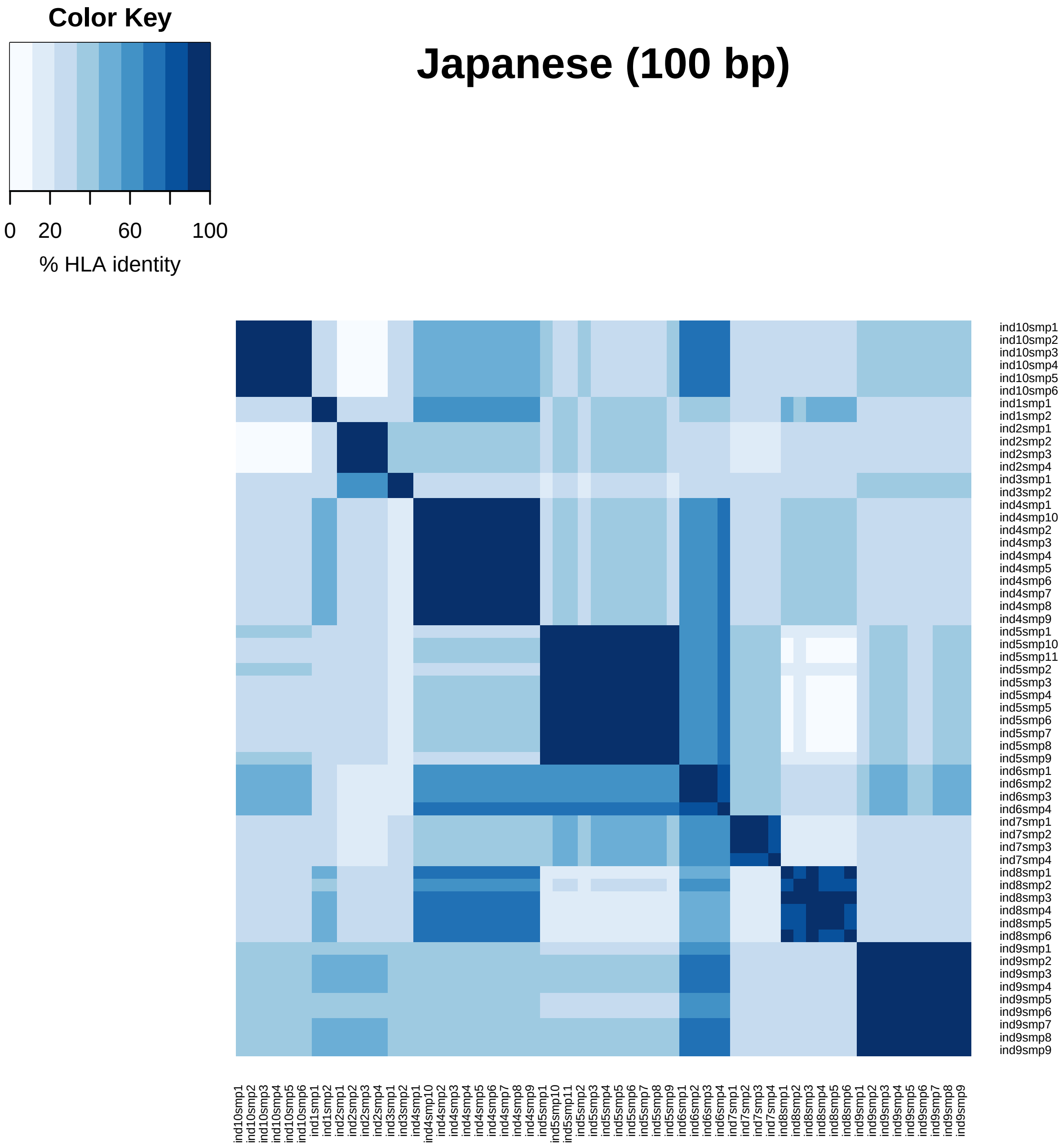
Color Key
0
20
60
100
% HLA identity
Japanese (100 bp)
ind10smp1
ind10smp2
ind10smp3
ind10smp4
ind10smp5
ind10smp6
ind1smp1
ind1smp2
ind2smp1
ind2smp2
ind2smp3
ind2smp4
ind3smp1
ind3smp2
ind4smp1
ind4smp10
ind4smp2
ind4smp3
ind4smp4
ind4smp5
ind4smp6
ind4smp7
ind4smp8
ind4smp9
ind5smp1
ind5smp10
ind5smp11
ind5smp2
ind5smp3
ind5smp4
ind5smp5
ind5smp6
ind5smp7
ind5smp8
ind5smp9
ind6smp1
ind6smp2
ind6smp3
ind6smp4
ind7smp1
ind7smp2
ind7smp3
ind7smp4
ind8smp1
ind8smp2
ind8smp3
ind8smp4
ind8smp5
ind8smp6
ind9smp1
ind9smp2
ind9smp3
ind9smp4
ind9smp5
ind9smp6
ind9smp7
ind9smp8
ind9smp9

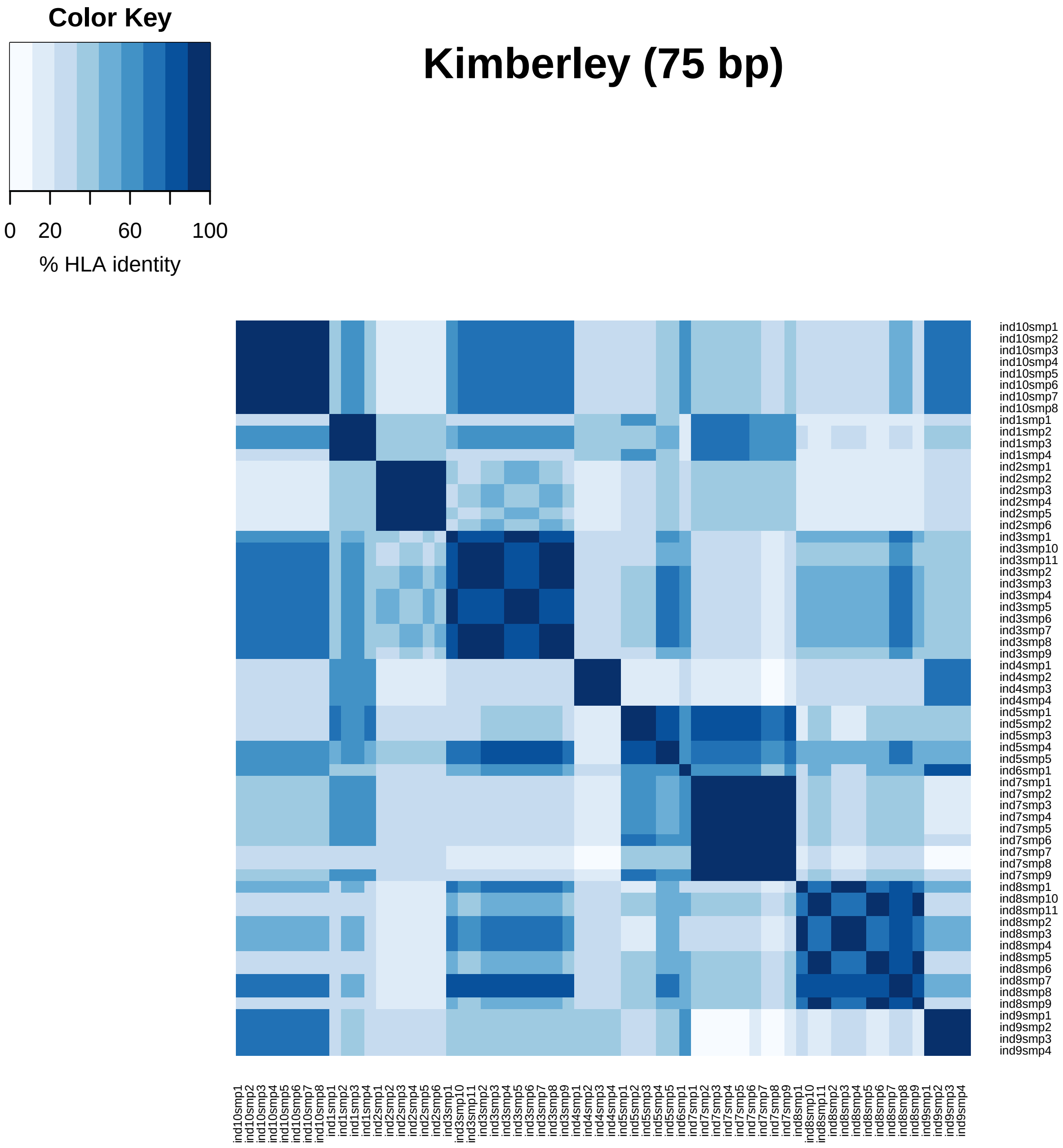
Color Key
0
20
60
100
% HLA identity
Kimberley (75 bp)
ind10smp1
ind10smp2
ind10smp3
ind10smp4
ind10smp5
ind10smp6
ind10smp7
ind10smp8
ind1smp1
ind1smp2
ind1smp3
ind1smp4
ind2smp1
ind2smp2
ind2smp3
ind2smp4
ind2smp5
ind2smp6
ind3smp1
ind3smp10
ind3smp11
ind3smp2
ind3smp3
ind3smp4
ind3smp5
ind3smp6
ind3smp7
ind3smp8
ind3smp9
ind4smp1
ind4smp2
ind4smp3
ind4smp4
ind5smp1
ind5smp2
ind5smp3
ind5smp4
ind5smp5
ind6smp1
ind7smp1
ind7smp2
ind7smp3
ind7smp4
ind7smp5
ind7smp6
ind7smp7
ind7smp8
ind7smp9
ind8smp1
ind8smp10
ind8smp11
ind8smp2
ind8smp3
ind8smp4
ind8smp5
ind8smp6
ind8smp7
ind8smp8
ind8smp9
ind9smp1
ind9smp2
ind9smp3
ind9smp4

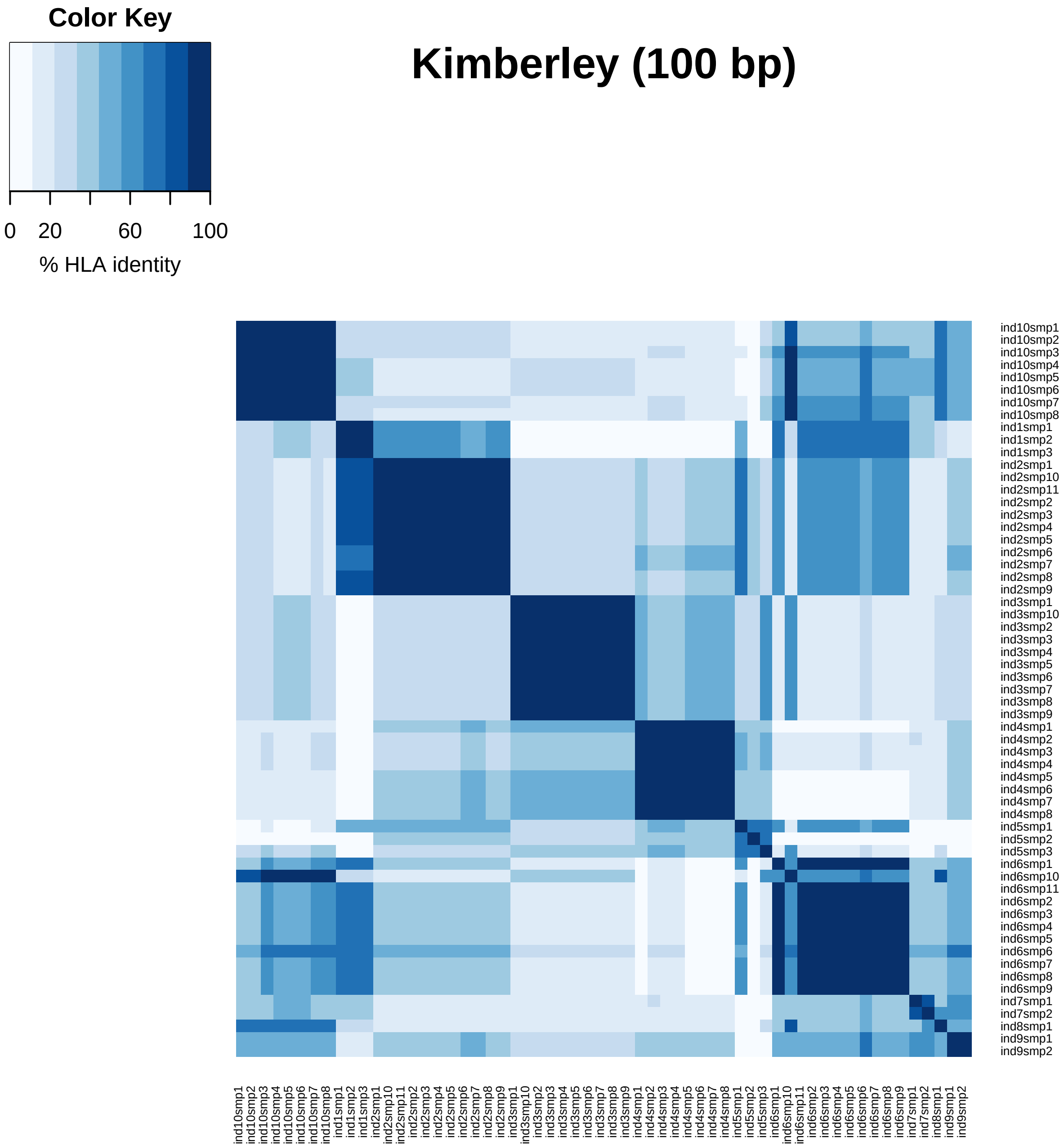

Color Key
0
20
60
100
% HLA identity
Kimberley (100 bp)
ind10smp1
ind10smp2
ind10smp3
ind10smp4
ind10smp5
ind10smp6
ind10smp7
ind10smp8
ind1smp1
ind1smp2
ind1smp3
ind2smp1
ind2smp10
ind2smp11
ind2smp2
ind2smp3
ind2smp4
ind2smp5
ind2smp6
ind2smp7
ind2smp8
ind2smp9
ind3smp1
ind3smp10
ind3smp2
ind3smp3
ind3smp4
ind3smp5
ind3smp6
ind3smp7
ind3smp8
ind3smp9
ind4smp1
ind4smp2
ind4smp3
ind4smp4
ind4smp5
ind4smp6
ind4smp7
ind4smp8
ind5smp1
ind5smp2
ind5smp3
ind6smp1
ind6smp10
ind6smp11
ind6smp2
ind6smp3
ind6smp4
ind6smp5
ind6smp6
ind6smp7
ind6smp8
ind6smp9
ind7smp1
ind7smp2
ind8smp1
ind9smp1
ind9smp2

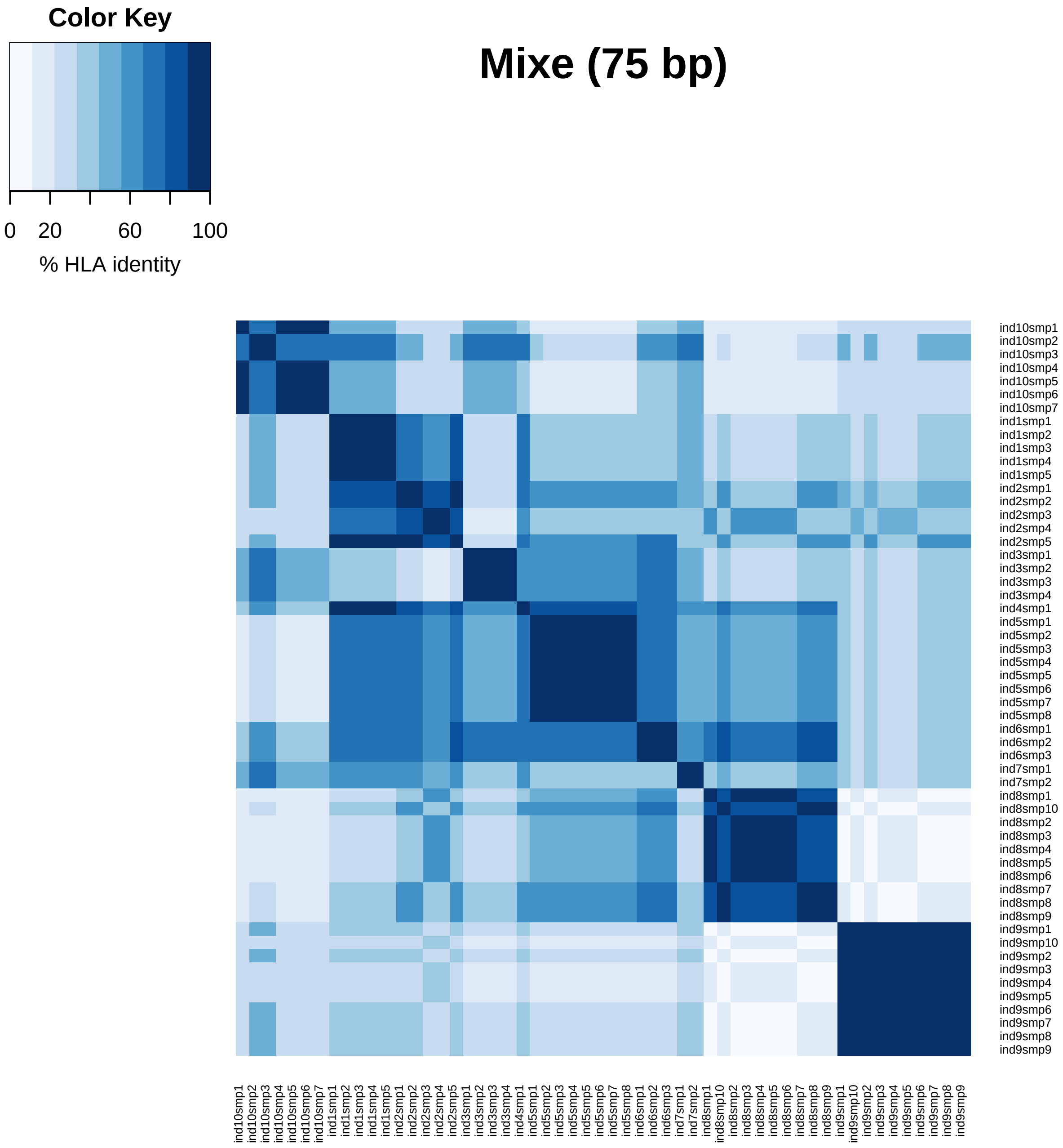
Color Key
0
20
60
100
% HLA identity
Mixe (75 bp)
ind10smp1
ind10smp2
ind10smp3
ind10smp4
ind10smp5
ind10smp6
ind10smp7
ind1smp1
ind1smp2
ind1smp3
ind1smp4
ind1smp5
ind2smp1
ind2smp2
ind2smp3
ind2smp4
ind2smp5
ind3smp1
ind3smp2
ind3smp3
ind3smp4
ind4smp1
ind5smp1
ind5smp2
ind5smp3
ind5smp4
ind5smp5
ind5smp6
ind5smp7
ind5smp8
ind6smp1
ind6smp2
ind6smp3
ind7smp1
ind7smp2
ind8smp1
ind8smp10
ind8smp2
ind8smp3
ind8smp4
ind8smp5
ind8smp6
ind8smp7
ind8smp8
ind8smp9
ind9smp1
ind9smp10
ind9smp2
ind9smp3
ind9smp4
ind9smp5
ind9smp6
ind9smp7
ind9smp8
ind9smp9

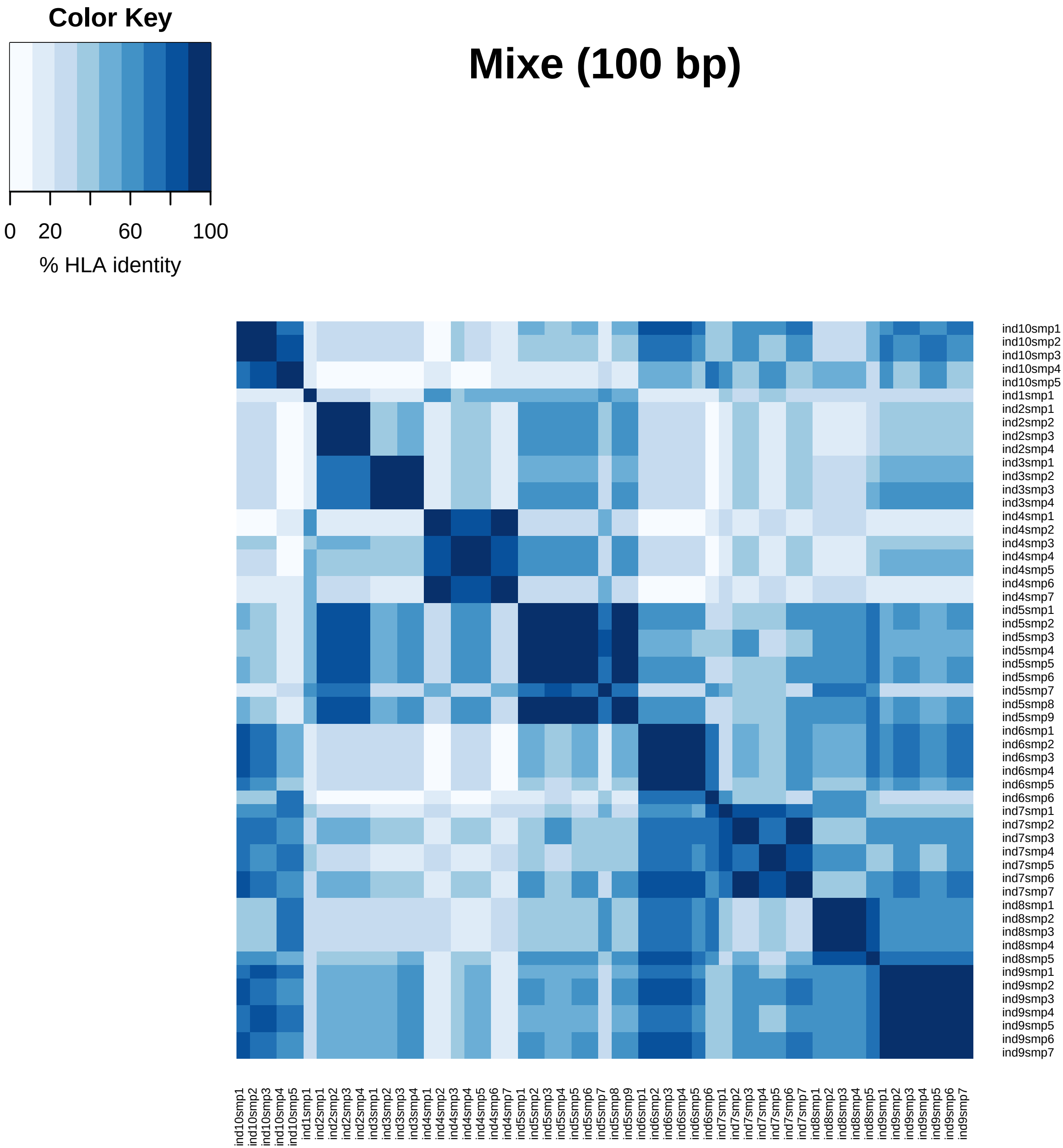

Color Key
0
20
60
100
% HLA identity
Mixe (100 bp)
ind10smp1
ind10smp2
ind10smp3
ind10smp4
ind10smp5
ind1smp1
ind2smp1
ind2smp2
ind2smp3
ind2smp4
ind3smp1
ind3smp2
ind3smp3
ind3smp4
ind4smp1
ind4smp2
ind4smp3
ind4smp4
ind4smp5
ind4smp6
ind4smp7
ind5smp1
ind5smp2
ind5smp3
ind5smp4
ind5smp5
ind5smp6
ind5smp7
ind5smp8
ind5smp9
ind6smp1
ind6smp2
ind6smp3
ind6smp4
ind6smp5
ind6smp6
ind7smp1
ind7smp2
ind7smp3
ind7smp4
ind7smp5
ind7smp6
ind7smp7
ind8smp1
ind8smp2
ind8smp3
ind8smp4
ind8smp5
ind9smp1
ind9smp2
ind9smp3
ind9smp4
ind9smp5
ind9smp6
ind9smp7

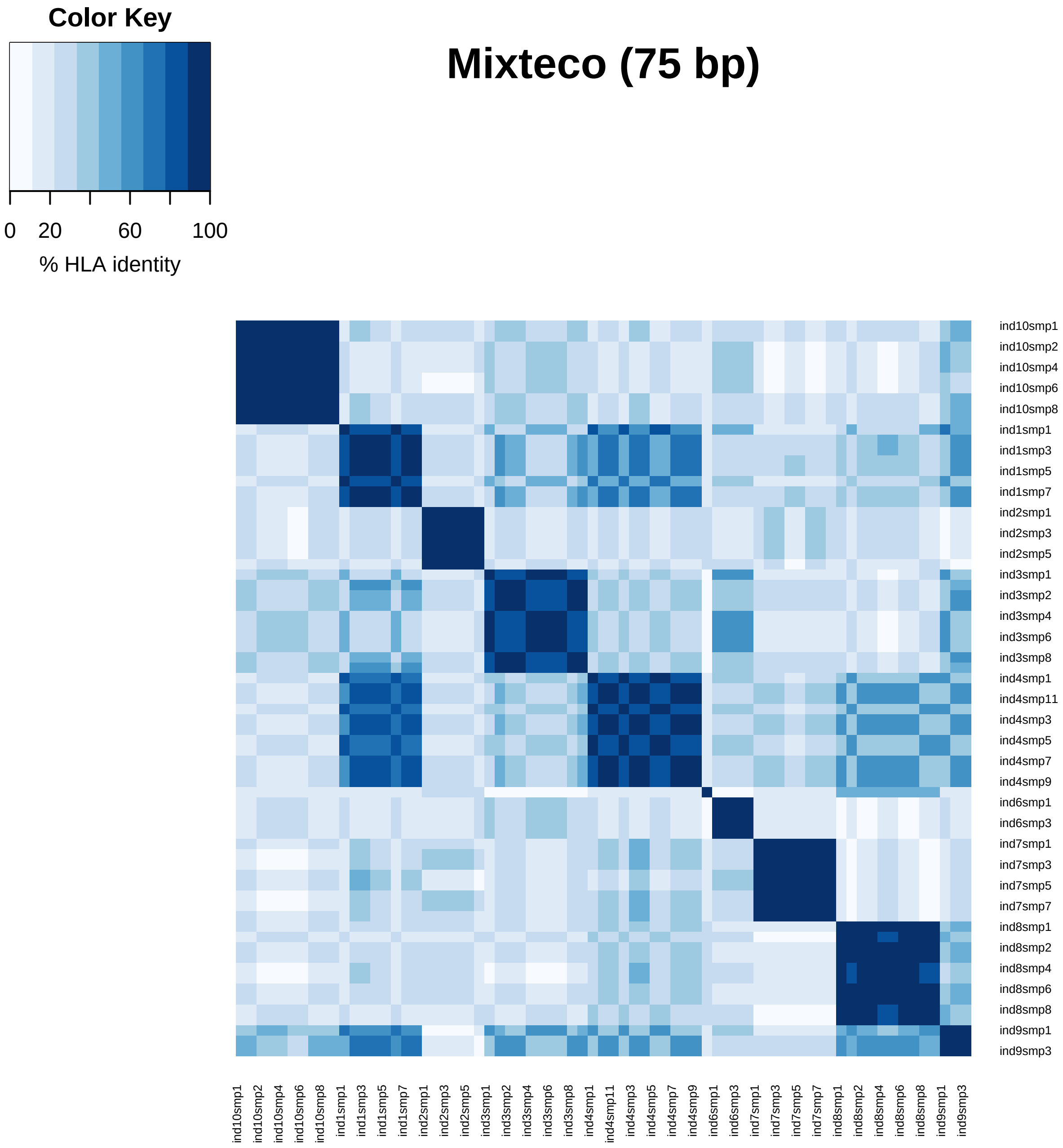
Color Key
0
20
60
100
% HLA identity
Mixteco (75 bp)
ind10smp1
ind10smp2
ind10smp4
ind10smp6
ind10smp8
ind1smp1
ind1smp3
ind1smp5
ind1smp7
ind2smp1
ind2smp3
ind2smp5
ind3smp1
ind3smp2
ind3smp4
ind3smp6
ind3smp8
ind4smp1
ind4smp11
ind4smp3
ind4smp5
ind4smp7
ind4smp9
ind6smp1
ind6smp3
ind7smp1
ind7smp3
ind7smp5
ind7smp7
ind8smp1
ind8smp2
ind8smp4
ind8smp6
ind8smp8
ind9smp1
ind9smp3
ind10smp1
ind10smp2
ind10smp4
ind10smp6
ind10smp8
ind1smp1
ind1smp3
ind1smp5
ind1smp7
ind2smp1
ind2smp3
ind2smp5
ind3smp1
ind3smp2
ind3smp4
ind3smp6
ind3smp8
ind4smp1
ind4smp11
ind4smp3
ind4smp5
ind4smp7
ind4smp9
ind6smp1
ind6smp3
ind7smp1
ind7smp3
ind7smp5
ind7smp7
ind8smp1
ind8smp2
ind8smp4
ind8smp6
ind8smp8
ind9smp1
ind9smp3

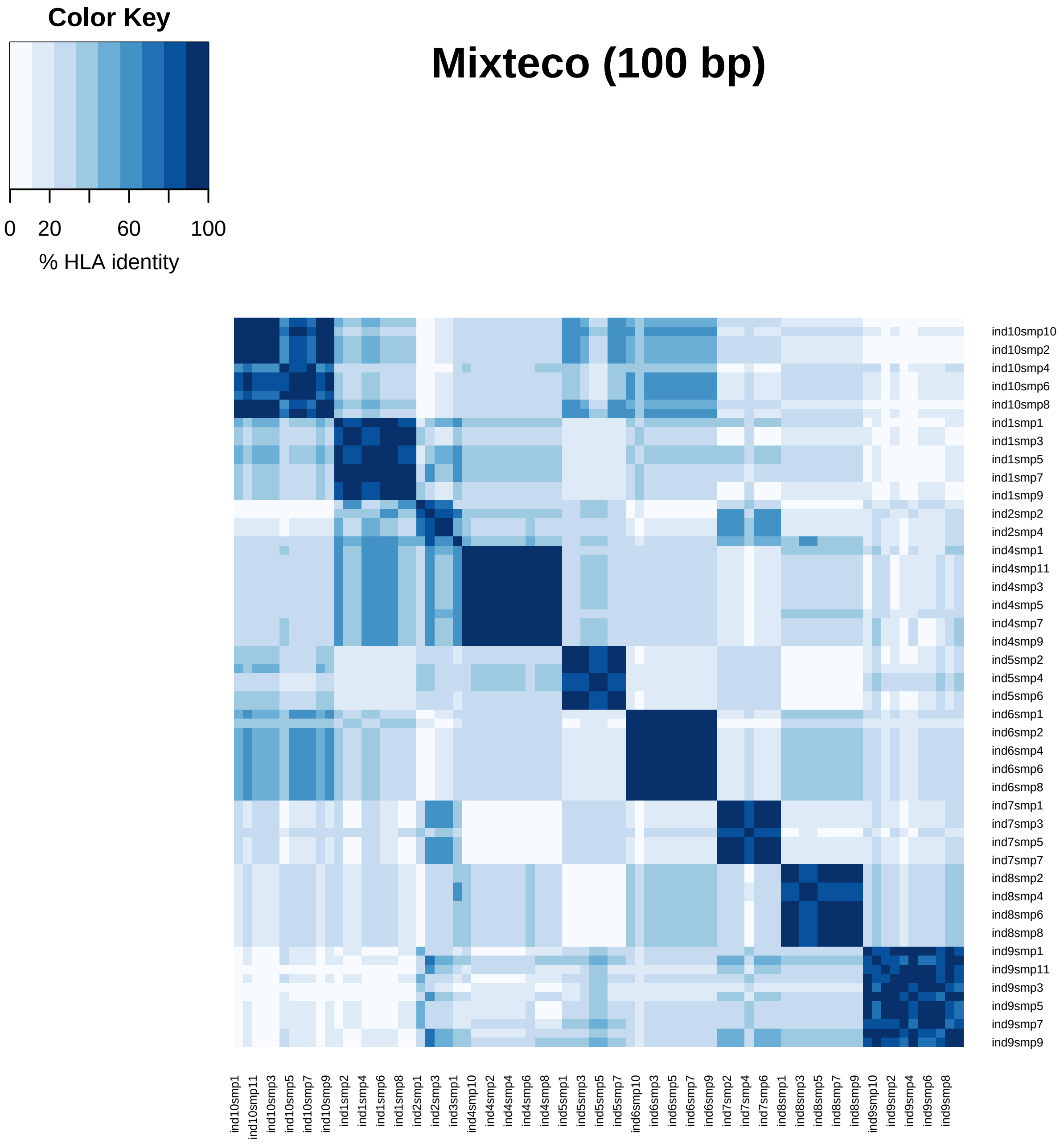
Color Key
0
20
60
100
% HLA identity
Mixteco (100 bp)
ind10smp10
ind10smp2
ind10smp4
ind10smp6
ind10smp8
ind1smp1
ind1smp3
ind1smp5
ind1smp7
ind1smp9
ind2smp2
ind2smp4
ind4smp1
ind4smp11
ind4smp3
ind4smp5
ind4smp7
ind4smp9
ind5smp2
ind5smp4
ind5smp6
ind6smp1
ind6smp2
ind6smp4
ind6smp6
ind6smp8
ind7smp1
ind7smp3
ind7smp5
ind7smp7
ind8smp2
ind8smp4
ind8smp6
ind8smp8
ind9smp1
ind9smp11
ind9smp3
ind9smp5
ind9smp7
ind9smp9
ind10smp1
ind10smp11
ind10smp3
ind10smp5
ind10smp7
ind10smp9
ind1smp2
ind1smp4
ind1smp6
ind1smp8
ind2smp1
ind2smp3
ind3smp1
ind4smp10
ind4smp2
ind4smp4
ind4smp6
ind4smp8
ind5smp1
ind5smp3
ind5smp5
ind5smp7
ind6smp10
ind6smp3
ind6smp5
ind6smp7
ind6smp9
ind7smp2
ind7smp4
ind7smp6
ind8smp1
ind8smp3
ind8smp5
ind8smp7
ind8smp9
ind9smp10
ind9smp2
ind9smp4
ind9smp6
ind9smp8

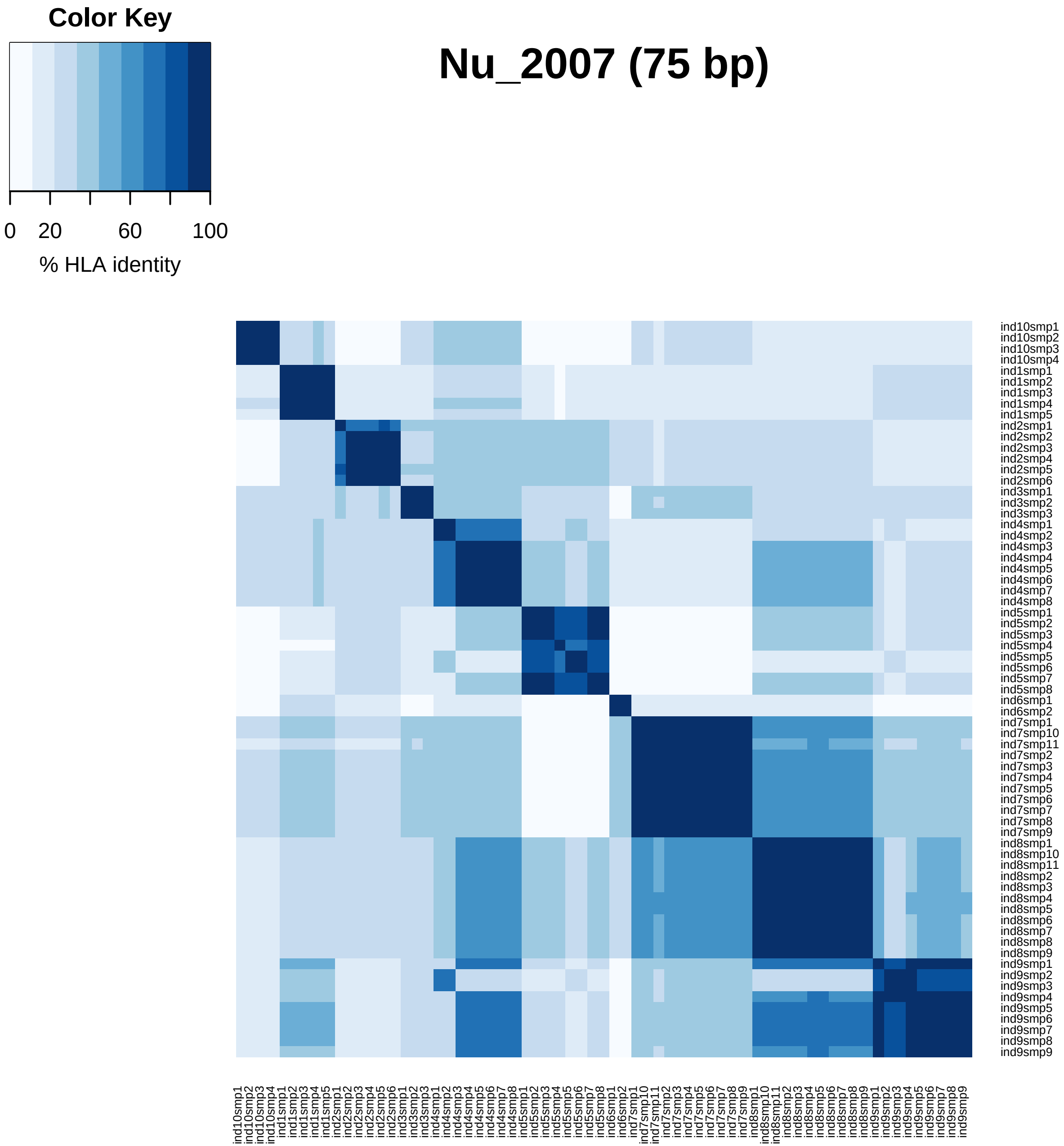
Color Key
0
20
60
100
% HLA identity
Nu_2007 (75 bp)
ind10smp1
ind10smp2
ind10smp3
ind10smp4
ind1smp1
ind1smp2
ind1smp3
ind1smp4
ind1smp5
ind2smp1
ind2smp2
ind2smp3
ind2smp4
ind2smp5
ind2smp6
ind3smp1
ind3smp2
ind3smp3
ind4smp1
ind4smp2
ind4smp3
ind4smp4
ind4smp5
ind4smp6
ind4smp7
ind4smp8
ind5smp1
ind5smp2
ind5smp3
ind5smp4
ind5smp5
ind5smp6
ind5smp7
ind5smp8
ind6smp1
ind6smp2
ind7smp1
ind7smp10
ind7smp11
ind7smp2
ind7smp3
ind7smp4
ind7smp5
ind7smp6
ind7smp7
ind7smp8
ind7smp9
ind8smp1
ind8smp10
ind8smp11
ind8smp2
ind8smp3
ind8smp4
ind8smp5
ind8smp6
ind8smp7
ind8smp8
ind8smp9
ind9smp1
ind9smp2
ind9smp3
ind9smp4
ind9smp5
ind9smp6
ind9smp7
ind9smp8
ind9smp9

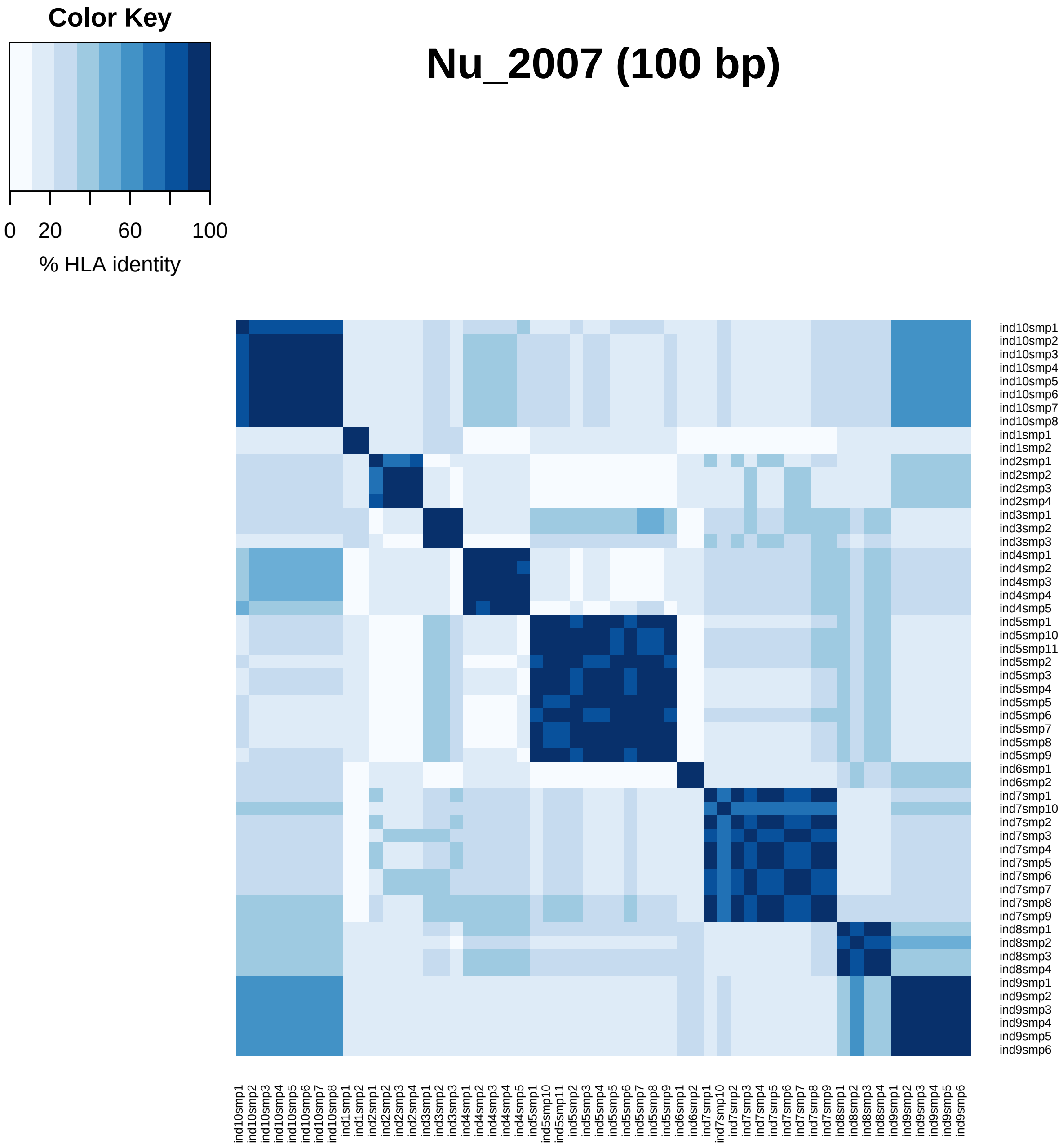
Color Key
0
20
60
100
% HLA identity
Nu_2007 (100 bp)
ind10smp1
ind10smp2
ind10smp3
ind10smp4
ind10smp5
ind10smp6
ind10smp7
ind10smp8
ind1smp1
ind1smp2
ind2smp1
ind2smp2
ind2smp3
ind2smp4
ind3smp1
ind3smp2
ind3smp3
ind4smp1
ind4smp2
ind4smp3
ind4smp4
ind4smp5
ind5smp1
ind5smp10
ind5smp11
ind5smp2
ind5smp3
ind5smp4
ind5smp5
ind5smp6
ind5smp7
ind5smp8
ind5smp9
ind6smp1
ind6smp2
ind7smp1
ind7smp10
ind7smp2
ind7smp3
ind7smp4
ind7smp5
ind7smp6
ind7smp7
ind7smp8
ind7smp9
ind8smp1
ind8smp2
ind8smp3
ind8smp4
ind9smp1
ind9smp2
ind9smp3
ind9smp4
ind9smp5
ind9smp6

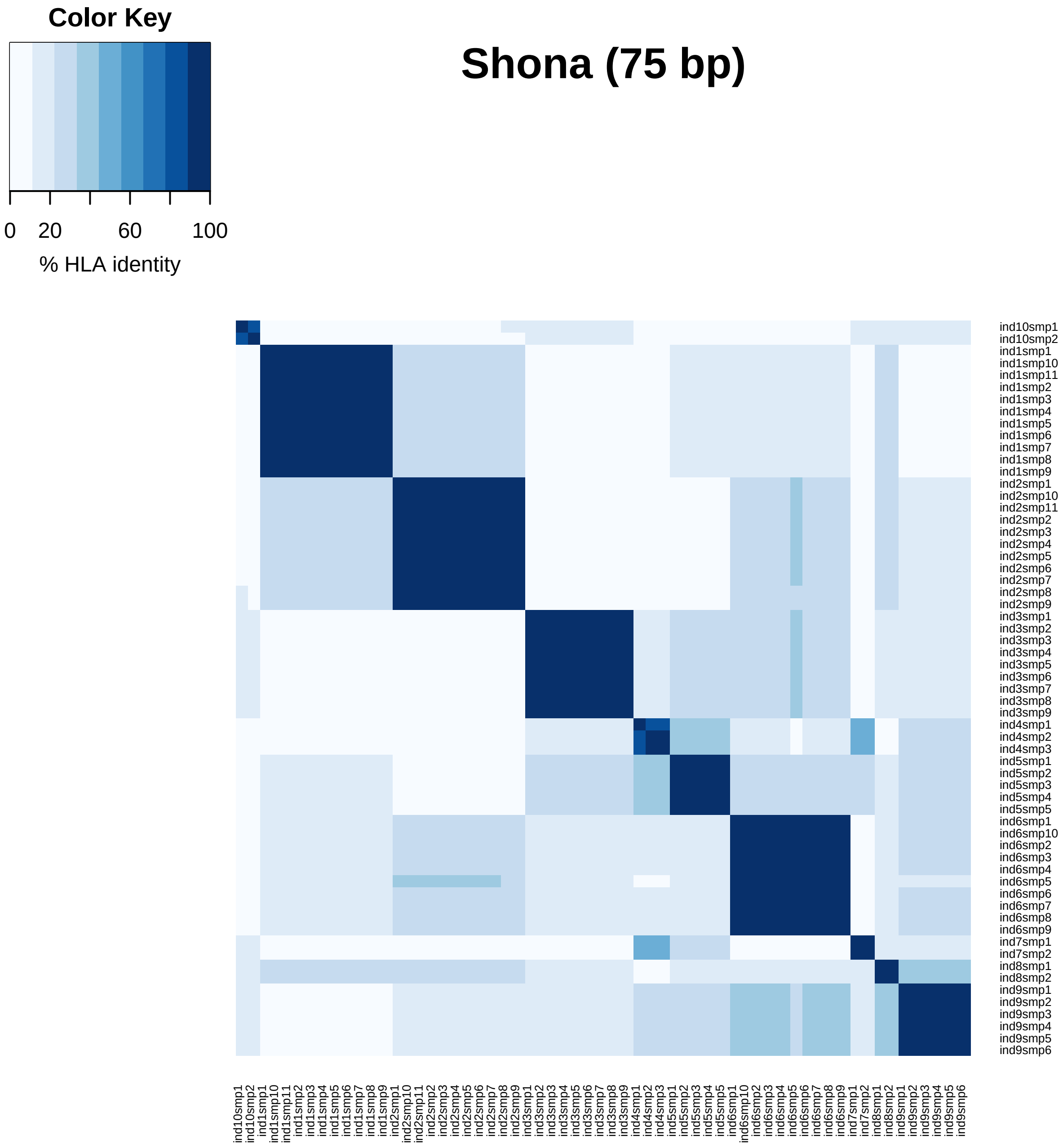
Color Key
0
20
60
100
% HLA identity
Shona (75 bp)
ind10smp1
ind10smp2
ind1smp1
ind1smp10
ind1smp11
ind1smp2
ind1smp3
ind1smp4
ind1smp5
ind1smp6
ind1smp7
ind1smp8
ind1smp9
ind2smp1
ind2smp10
ind2smp11
ind2smp2
ind2smp3
ind2smp4
ind2smp5
ind2smp6
ind2smp7
ind2smp8
ind2smp9
ind3smp1
ind3smp2
ind3smp3
ind3smp4
ind3smp5
ind3smp6
ind3smp7
ind3smp8
ind3smp9
ind4smp1
ind4smp2
ind4smp3
ind5smp1
ind5smp2
ind5smp3
ind5smp4
ind5smp5
ind6smp1
ind6smp10
ind6smp2
ind6smp3
ind6smp4
ind6smp5
ind6smp6
ind6smp7
ind6smp8
ind6smp9
ind7smp1
ind7smp2
ind8smp1
ind8smp2
ind9smp1
ind9smp2
ind9smp3
ind9smp4
ind9smp5
ind9smp6

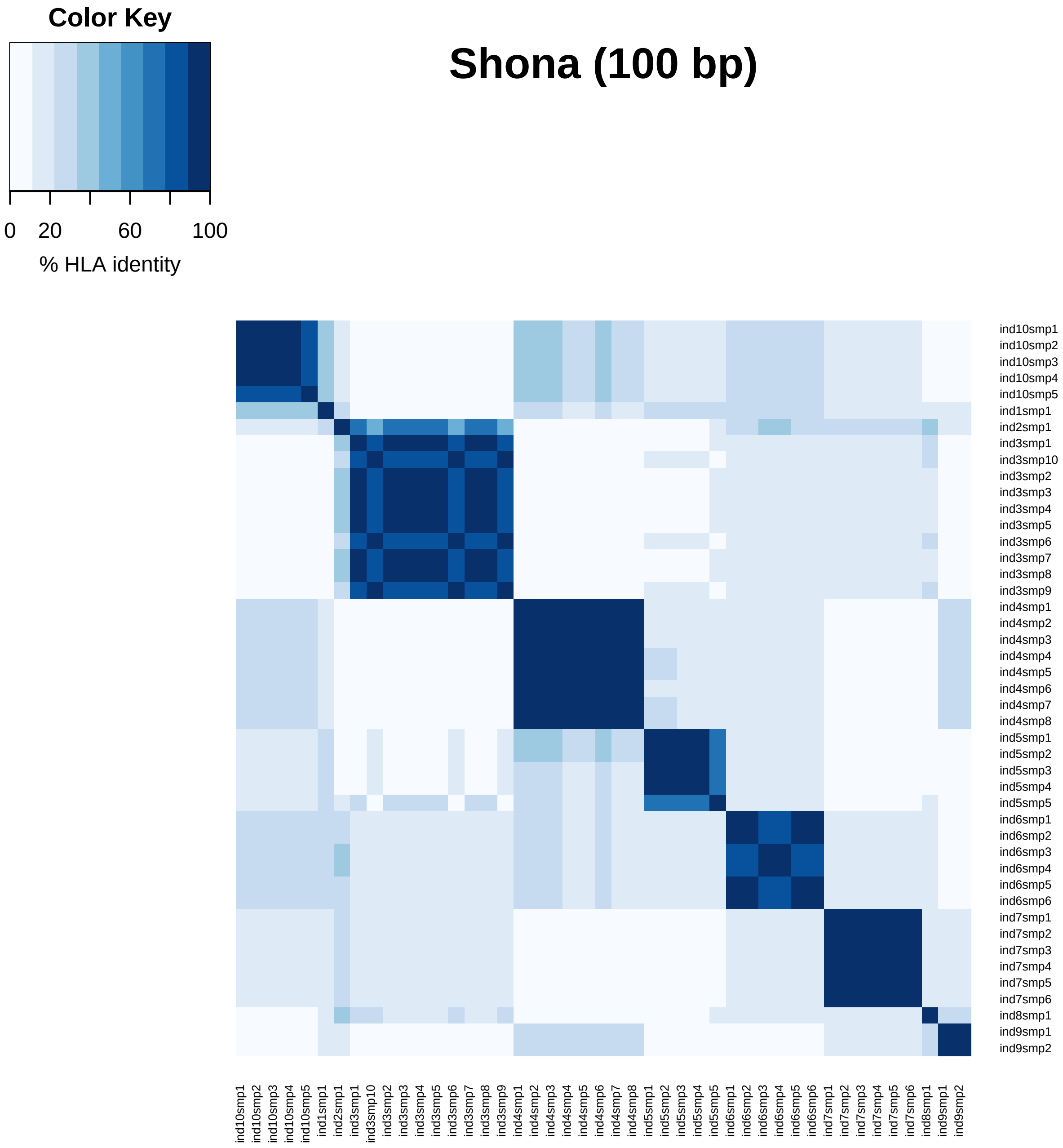

Color Key
0
20
60
100
% HLA identity
Shona (100 bp)
ind10smp1
ind10smp2
ind10smp3
ind10smp4
ind10smp5
ind1smp1
ind2smp1
ind3smp1
ind3smp10
ind3smp2
ind3smp3
ind3smp4
ind3smp5
ind3smp6
ind3smp7
ind3smp8
ind3smp9
ind4smp1
ind4smp2
ind4smp3
ind4smp4
ind4smp5
ind4smp6
ind4smp7
ind4smp8
ind5smp1
ind5smp2
ind5smp3
ind5smp4
ind5smp5
ind6smp1
ind6smp2
ind6smp3
ind6smp4
ind6smp5
ind6smp6
ind7smp1
ind7smp2
ind7smp3
ind7smp4
ind7smp5
ind7smp6
ind8smp1
ind9smp1
ind9smp2
